# Tetrazine Glycoconjugate for Pretargeted Positron Emission Tomography Imaging of *trans*-Cyclooctene-Functionalized Molecular Spherical Nucleic Acids

**DOI:** 10.1021/acsomega.3c04041

**Published:** 2023-11-24

**Authors:** Tatsiana Auchynnikava, Antti Äärelä, Heidi Liljenbäck, Juulia Järvinen, Putri Andriana, Luciana Kovacs, Jarkko Rautio, Johan Rajander, Pasi Virta, Anne Roivainen, Xiang-Guo Li, Anu J. Airaksinen

**Affiliations:** † Turku PET Centre, University of Turku, Turku FI-20520, Finland; ‡ Department of Chemistry, University of Turku, Turku FI-20500, Finland; § Research and Development, Orion Pharma, Turku FI-20380, Finland; ∥ Turku Center for Disease Modeling, University of Turku, Turku FI-20520, Finland; ⊥ School of Pharmacy, University of Eastern Finland, Kuopio FI-70210, Finland; # Accelerator Laboratory, Åbo Akademi University, Turku FI-20520, Finland; ∇ InFLAMES Research Flagship Center, University of Turku, Turku FI-20520, Finland

## Abstract

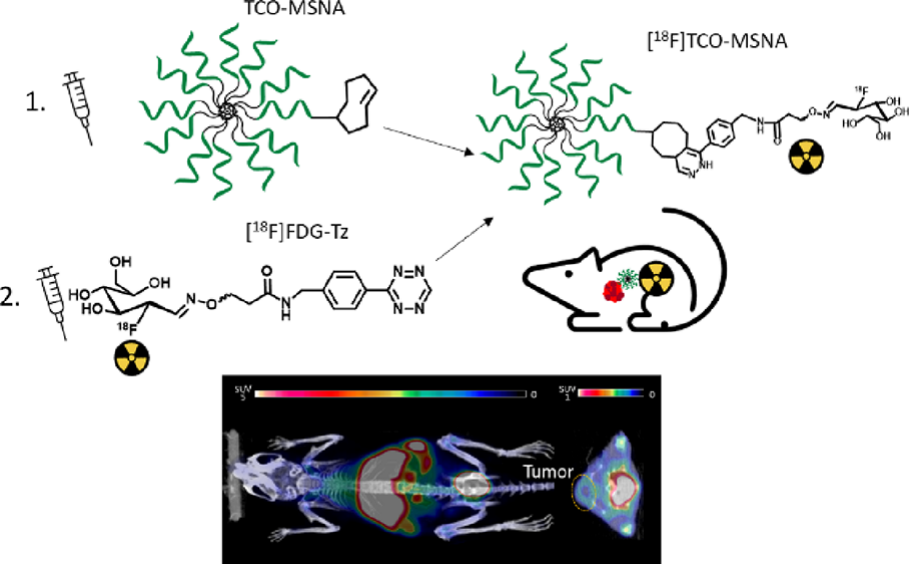

Pretargeted concept in positron emission tomography (PET) together with bioorthogonal chemistry is an elegant solution to study processes with slow pharmacokinetics by utilizing radiotracers labeled with short-lived radionuclides. Namely, radiotracers based on tetrazine ligation with *trans*-cyclooctene (TCO) via the inverse electron demand Diels–Alder (IEDDA) reaction have become a state-of-the-art for the pretargeted PET imaging. For radiolabeling of tetrazine scaffolds, indirect radiofluorination methods are often preferred, as tetrazines are vulnerable to harsh conditions typically necessary for the direct radiofluorination. ^18^F-Fluoroglycosylation is an indirect radiofluorination method, which allows the introduction of a widely accessible glucose analog 2-[^18^F]fluoro-2-deoxy-d-glucose ([^18^F]FDG) to aminooxy-functionalized precursors via oxime formation. Here, we report the biological evaluation of [^18^F]FDG-Tz as a tracer for pretargeted PET imaging of TCO-functionalized molecular spherical nucleic acids (MSNA) against human epidermal growth factor receptor 2 (HER2) mRNA. The oxime ether formation between [^18^F]FDG and tetrazine oxyamine resulted in [^18^F]FDG-Tz with high radiochemical purity (>99%) and moderate yields (6.5 ± 3.6%, *n* = 5). Biological evaluation of [^18^F]FDG-Tz in healthy mice indicated favorable pharmacokinetics with quick blood clearance, urinary excretion as the main elimination route, and the absence of GLUT1 transportation. The successful pretargeted experiments with TCO-functionalized MSNA revealed higher tumor uptake compared to preclicked MSNA in HER2-expressing human breast cancer xenograft-bearing mice.

## Introduction

Positron emission tomography (PET) is a sensitive noninvasive molecular imaging technique, which is used in clinical diagnostics, disease monitoring, and drug development. In drug development, PET is an important tool for the investigation of pharmacokinetics of new drug candidates in early clinical studies and dose optimization studies.^1−3^ Since PET is based on the administration of radiotracers, the half-life of the radionuclide must be compatible with the pharmacokinetics of the drug candidate. There are several factors that affect drug pharmacokinetics, and one of them is the size—large biomolecules, such as antibodies and other large proteins, typically have long circulation half-lives in the blood, slowing down their accumulation at the target site.^4,5^ The same is true with some nanomaterials, which may circulate even for a few days.^6,7^ Using nuclear imaging for tracing drug candidates with slow pharmacokinetics requires radionuclides with long half-lives, which may cause an increased radiation burden to the patient.^8,9^

**Figure 1:**
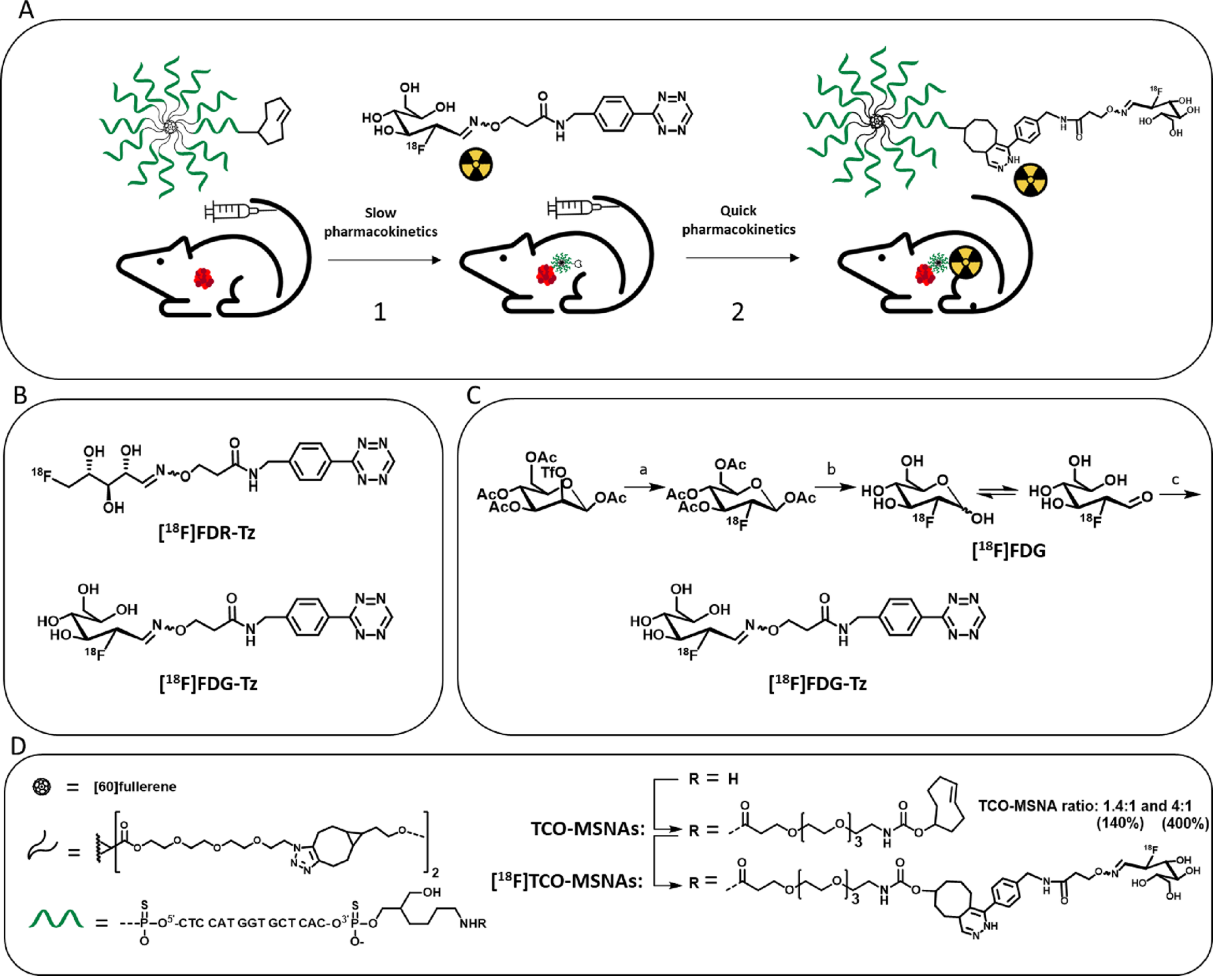
(A) The principle of pretargeted PET imaging is based on the IEDDA reaction. As the first step, the targeting agent (e.g., TCO-modified nanomaterial) is administrated. After giving it sufficient time to target the tumor and clear from the blood and healthy tissues, the radiotracer (e.g., radiolabeled tetrazine derivative) is administrated, which immediately reacts with the TCO moiety. The excess of the radiotracer rapidly clears from the blood. (B) Chemical structures of [^18^F]FDR conjugated with aminooxy-functionalized tetrazine ([^18^F]FDR-Tz) and [^18^F]FDG conjugated with aminooxy-functionalized tetrazine ([^18^F]FDG-Tz) investigated in this study. (C) Synthesis of [^18^F]FDG-Tz. Reagents and conditions: (a) K[^18^F]F–K222, K_2_CO_3_, CH_3_CN, thexyl alcohol, 85 °C, 5 min, (b) 2 M NaOH, (c) tetrazine oxyamine, anilinium acetate-buffer pH 4.6, CH_3_CN, MeOH, 75 °C, 30 min. (D) Structural details of [^18^F]TCO-MSNAs (TCO-modified MSNA radiolabeled with [^18^F]FDG-Tz).

One recently investigated solution for this is to utilize bioorthogonal chemistry and a pretargeted approach for radiolabeling the targeting agent (i.e., the drug candidate) *in vivo* with a separately administered diagnostic radiotracer after the targeting agent has already accumulated to its target site. This is called pretargeted imaging and is a concept that enables to study processes with slow kinetics by using short-lived radionuclides. Tetrazine ligation, a cutting-edge method involving the inverse electron demand Diels–Alder (IEDDA) reaction between tetrazine and trans-cyclooctene (TCO), followed by the retro-Diels–Alder reaction, is a leading approach in pretargeted PET imaging. This technique is prized for its instantaneous reaction rates, biocompatibility, versatility, and chemoselectivity.^10−12^ The approach allows sufficient time, which can range from hours to days, for the targeting moiety to accumulate in the desired tissue and to clear from the circulation before applying the bioorthogonal radiotracer for radiolabeling the moiety.^13−17^ The introduction of a two-step approach not only greatly lowers radiation dose to healthy tissue but also improves PET image contrast by decreasing the background radioactivity in nontarget organs (Figure 1A).^11,18^

A range of targeting vectors, such as antibodies^15,17,19,20^ and other nanomedicines^7,16,21,22^ modified with TCO have been radiolabeled *in vivo* with various radiolabeled tetrazine derivatives to investigate the pretargeting concept. Despite all the benefits of the pretargeting approach, the development of new efficient PET radiotracers is still needed for the adaptation of this multistep strategy to daily clinical use. Pharmacokinetics of tetrazine affects the success of the approach and finding an optimal tetrazine derivative for efficient IEDDA reaction *in vivo* is not an effortless task. To minimize background signal, the radiolabeled tetrazine should be quickly eliminated via a favorable elimination route but still circulate long enough to react with the TCO. Furthermore, the balance between the reactivity of the compound and its *in vivo* stability needs to be taken into consideration.^23,24^

For clinical applications, radiolabeling with fluorine-18 is a preferable choice due to its low β^+^ energy (maximum 0.64 MeV) and suitable physical half-life (109.8 min).^27^ Unfortunately, tetrazine scaffolds are vulnerable to harsh conditions of direct ^18^F-fluorination, like high pH and temperature, so many classical direct radiofluorination methods cannot be applied. Nevertheless, a variety of ^18^F-radiolabeled tetrazines have been reported, many of them synthesized via indirect methods by using ^18^F-fluorinated prosthetic groups.^10,25,28−31^ One of the indirect methods is ^18^F-fluoroglycosylation via oxime ether formation, which has been successfully utilized to introduce carbohydrate moiety to tetrazine derivatives.^25,26,32^ It is not only a chemoselective, mild labeling method, which can be carried out in aqueous solutions, but also a way to improve *in vivo* blood stability and kinetics and bioavailability and accelerate the clearance.^33^

We have previously reported the synthesis of [^18^F]FDR-Tz and [^18^F]FDG-Tz from two different [^18^F]fluorinated carbohydrates, 5-[^18^F]fluoro-5-deoxyribose and 2-[^18^F]fluoro-2-deoxy-d-glucose, via conjugation with aminooxy-functionalized tetrazine; however, only [^18^F]FDR-Tz was evaluated for pretargeted imaging (Figure 1B).^25,26^ The preclinical evaluation of [^18^F]FDR-Tz demonstrated that it has favorable pharmacokinetics, including optimal urinary elimination and an acceptable blood circulation time. However, some hepatobiliary excretion was observed for [^18^F]FDR-Tz, indicating moderate liver uptake 60 min after tracer administration. In addition, the consequently elevated radioactivity in the small and large intestines challenges the quantification of the nearby organs. Encouraged by the discovered benefits and challenges, we decided to evaluate also the [^18^F]FDG-conjugate, aiming to overcome the observed issues and even further improve the pharmacokinetic properties of the radiolabeled tetrazine. [^18^F]FDG is considered to be the gold standard for PET imaging and is widely accessible. Its use is based on the transport, especially via glucose transporters GLUT1 and GLUT3 in metabolically active cancer cells, and phosphorylation by hexokinase and subsequent accumulation inside the cell.^34,35^ Commercially available [^18^F]FDG has a high glucose concentration, which hampers its use as a prosthetic group for the synthesis of oxime-conjugates with high molar activity without removal of the residual glucose. Nevertheless, it is an expansively available starting material and [^18^F]FDG has been successfully applied as a prosthetic group for peptide radiolabeling.^27,36−40^ The previous studies have proved ^18^F-fluoroglycosylation as a chemoselective, effective, and simple method. Furthermore, the carbohydrate introduction has shown to decrease peptides’ lipophilicity, improving the *in vivo* pharmacokinetic profile and leading toward renal excretion over hepatobiliary excretion. Moreover, along with beneficial metabolic stability, it may improve tumor uptake.^27,38,40^ Radiochemical yields (RCYs) of ^18^F-fluoroglycosylation are dependent on the mutarotation of [^18^F]FDG in aqueous solution with the dominance of β–pyranose over the intermediate acyclic aldehyde form. Higher temperatures and acidic conditions have been shown to stimulate the ^18^F-fluoroglycosylation.^27^

Spherical nucleic acids (SNAs) are highly oriented nanostructures. Their building blocks include suitable nanoparticle core unit (gold, quantum dots, silica, liposomes, proteins, polymers), layered with radially oriented densely packed oligonucleotides (ONs).^41−43^ Due to singular structure, SNAs demonstrate beneficial characteristics over linear ONs they are derived from, namely, more efficient cellular uptake due to good ability to cross different biological barriers (e.g., blood-brain barrier, blood-tumor barrier, and skin) via scavenger receptor-mediated endocytosis, lower immune response, and higher resistance to nuclease degradation.^44−46^ As a result, SNAs have great potential as delivery vehicles to transport a large amount of bioactive material. Recently, the controlled monofunctionalization of [60]fullerene-based molecular SNA (MSNA) with ^68^Ga-radiolabeling was reported. The presented MSNA consisted of an anti-HER2 (human epidermal growth factor receptor 2) ON sequence, which can regulate HER2 protein expression.^47^ HER2 is overexpressed in 15–20% of breast cancer cases.^48^ By functionalizing MSNA with TCO, we open the perspective for tracing the MSNA biodistribution by pretargeted PET imaging based on the *in vivo* IEDDA reaction with tetrazine derivatives.

Here, we conducted synthesis and biological evaluation of [^18^F]FDG-Tz and *in vitro* in human oral squamous carcinoma CAL27 cell line and in healthy mice, followed by pretargeted PET imaging of TCO-functionalized MSNA (Figure 1C) in HER2-expressing human breast cancer HCC1954 tumor xenografts-bearing mice by using [^18^F]FDG-Tz.

## Result and Discussion

### Radiosynthesis of [^18^F]FDG-Tz

[^18^F]FDG-Tz was synthesized in two steps as depicted in Scheme S1. [^18^F]FDG was obtained via nucleophilic [^18^F]fluorination from tetra-acetylated mannose triflate. Compared to our previously reported method,^26^ the use of thexyl alcohol as a solvent for this step allowed to increase [^18^F]fluorination yields from 69% up to 92%, determined by radio-thin-layer chromatography (TLC) of the reaction mixture.^49^ To prevent competing reactions in oxime formation, the excess of the precursor was removed from the tetra-acetylated [^18^F]FDG solution prior to the deprotection with semi-preparative high-performance liquid chromatography (HPLC). Acidic conditions and aniline catalysis can accelerate the reaction between aminooxy and carbonyl groups,^27^ so freshly prepared anilinium acetate buffer with pH 4.6 was used. Additionally, to facilitate the conjugation reaction, methanol was added to the reaction, and the temperature was increased from 60 to 75 °C. These improved the reaction yield from 5 to 40%. To remove unreacted tetrazine precursor, the second semi-preparative HPLC was conducted.

RCY and molar activity of the final isolated product [^18^F]FDG-Tz were 6.5% ± 3.6 and 14.7 ± 4.0 GBq/μmol (*n* = 5), respectively. RCY was decay-corrected to the end of bombardment (EOB), and molar activity refers to the end of synthesis (EOS). The radiochemical purity at EOS was >99% and remained >98% for 6 h from EOS (longer time was not tested). The total duration of radiosynthesis was 3 h and 5 min starting from the EOB. Figure S1 in the Supporting Information represents the results of typical quality control of the final purified [^18^F]FDG-Tz including radio-HPLC and radio-TLC. As reported earlier, radio-HPLC detects three product peaks for [^18^F]FDG-Tz, which are also confirmed by our studies. These peaks represent the acyclic *E–* and *Z–* isomers and the cyclic pyranose isomer.^26^

### GLUT1 Binding Affinity and Cellular Uptake

**Figure 2:**
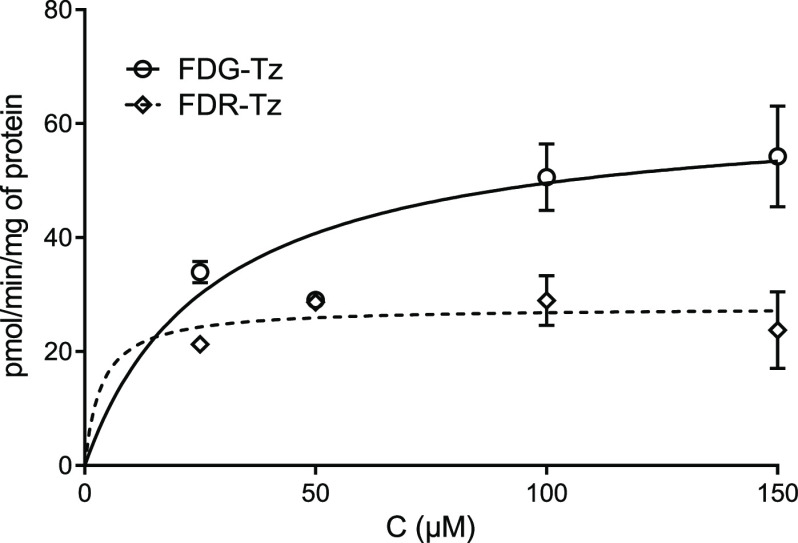
Cellular uptake of compounds FDR-Tz and FDG-Tz in the CAL27 cell line after incubation in the 5–400 μM range for 5 min. Michaelis–Menten kinetic parameters (when available) for FDG-Tz: *V*_max_ (pmol/min/mg of protein) = 63.2 ± 8.5 and *K*_m_ (μM) = 27.6 ± 13.8; for FDR-Tz: *V*_max_ (pmol/min/mg of protein) = 27.8 ± 3.7 and *K*_m_ (μM) = 3.6 ± 7.8.

Introduction of the [^18^F]FDG as a prosthetic radiolabeling group may cause an increase in nonspecific accumulation of the studied structure due to GLUT1 binding. Therefore, to confirm or refute the transportation of [^18^F]FDG-Tz via GLUT1, binding affinity and cellular uptake were analyzed in CAL27 cells and compared to the corresponding ribose derivative, [^18^F]FDR-Tz. Half-maximal inhibitory concentration (IC_50_) for FDG-Tz was 324.4 μM and for FDR-Tz it was 22.4 μM, indicating that FDR-Tz has almost 15 times higher affinity for GLUT1 compared to FDG-Tz. For d-glucose IC_50_ > 1 mM has been reported in CAL27 cells.^50^ Cellular uptake studies showed that FDG-Tz is transported into cells slightly more efficiently compared to FDR-Tz (*V*_max_ = 63.2 ± 8.5 pmol/min/mg of protein and *K*_m_ = 27.6 ± 13.8 μM for compound FDG-Tz; *V*_max_ = 27.8 ± 3.7 pmol/min/mg of protein and *K*_m_ = 3.6 ± 7.8 μM for compound FDR-Tz). From the performed studies, we can conclude that FDG-Tz is transported more efficiently, although it has lower GLUT1 affinity compared to FDR-Tz. The shape of the curve (Figure 2) also supports this interpretation, as FDG-Tz uptake is concentration-dependent, while FDR-Tz is not. However, for the extracellularly targeting imaging agent, GLUT1 transportation is a concerning and disadvantaging feature.

### [^18^F]FDG-Tz Radioactivity Distribution in Blood Components

**Figure 3:**
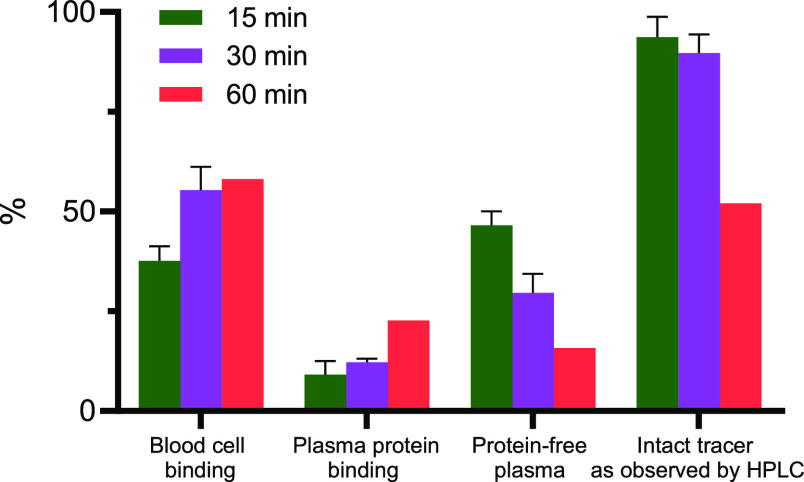
Measurement of [^18^F]FDG-Tz radioactivity concentration in blood components at 15 (*n* = 3), 30 (*n* = 3), and 60 min (*n* = 2) postinjection revealed an increase in blood cell binding and plasma protein binding and a decrease in intact tracer over time.

Analysis of [^18^F]FDG-Tz radioactivity distribution in different blood components of healthy mice showed an increase in blood cell binding from 37.6% ± 3.0 (*n* = 3) to 58.1% (*n* = 2) and in plasma protein binding from 9.1% ± 3.5 (*n* = 3) to 22.7% (*n* = 2) from 15 to 60 min postinjection (Figure 3). The increase can be related to the observed [^18^F]FDG-Tz GLUT1 affinity or its radioactive metabolites, as this transporter is widely expressed in erythrocytes.^51^ Radiometabolite analysis of [^18^F]FDG-Tz revealed that the proportion of intact tracer in plasma decreased from 93.7% ± 5.1 (*n* = 3) to 52% (*n* = 1) over 60 min.

### *Ex Vivo* Evaluation

**Figure 4:**
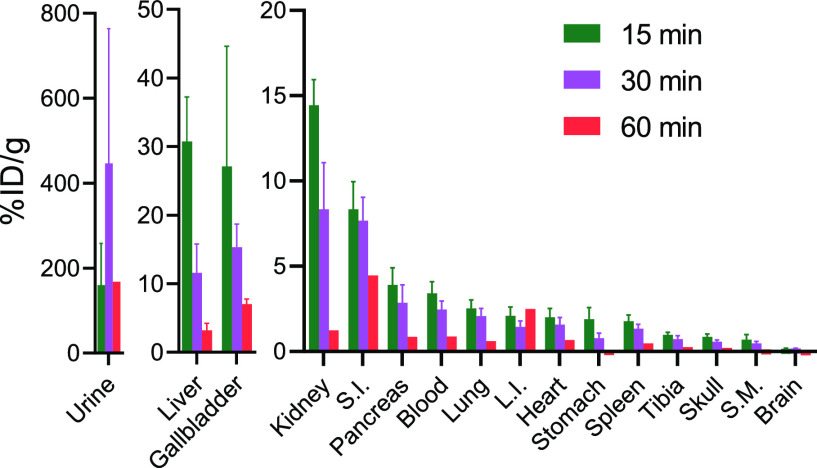
[^18^F]FDG-Tz *ex vivo* biodistribution after i.v. administration in healthy mice at 15 (*n* = 6), 30 (*n* = 4), and 60 min (*n* = 2) postinjection, demonstrating quick tracer elimination from the blood (S.M., skeletal muscle; S.I., small intestine; L.I., large intestine).

*Ex vivo* biodistribution of [^18^F]FDG-Tz was investigated in healthy female Balb/cAnNrj mice at three time points after intravenous (i.v.) administration of the tracer (1.1–11.4 MBq in 50–170 μL) via the lateral tail vein (Figure 4). The tissue weight normalized radioactivity concentration (%ID/g) indicates the major urinary type of excretion with minor elimination via the hepatobiliary route as can be seen from the moderate gallbladder radioactivity at 60 min (7.0 ± 0.7%ID/g). Despite the observed binding to blood cells, the tracer quickly eliminates from blood, leaving only %ID/g 0.9 ± 0.3 at 60 min, and exhibits moderate tracer accumulation in the liver and kidneys (3.2 ± 1.0 and 1.2 ± 0.4, respectively). Low brain uptake (0.08 ± 0.03) at 60 min postinjection indicates low penetration through the blood-brain barrier. Mainly low lipophilicity for pretargeted PET imaging candidates is desirable to avoid higher liver uptake and nonspecific binding and facilitate the IEDDA reaction.^25,52^ Furthermore, Herth’s group has reported that for crossing the blood-brain-barrier, compounds need to have a slow metabolism and high lipophilicity.^53^

The overall observed low uptake of [^18^F]FDG-Tz in GLUT1-rich tissues indicates the absence of GLUT1 transportation *in vivo*.^54,55^ In addition, [^18^F]FDG-Tz exhibits excellent *in vivo* stability and absence of defluorination, as %ID/g for tibia bone is just 0.26 ± 0.04 at 60 min postinjection.

As GLUT1 transportation is affected by glucose, fasting has been reported to affect organ accumulation of [^18^F]FDG. Thus, we decided to investigate if fasting affects biodistribution of [^18^F]FDG-Tz or not. Firmly, the statistical analysis showed no statistically significant difference between fasted and nonfasted animals 15 min postinjection for [^18^F]FDG-Tz (Figure S2), bracing the sparse effect of GLUT1 transportation on [^18^F]FDG-Tz. However, notable variation was observed between individual nonfasted animals in the gallbladder and urine. In addition, [^18^F]FDG biodistribution is known for being dramatically affected not only by animal dietary conditions but their handling as well.^56,57^ As shown in Figure S3, there was a trend of lower accumulation of [^18^F]FDG-Tz in several organs in awake animals after tracer administration compared to that in those anesthetized with isoflurane throughout the period between injection and sacrifice.

### [^18^F]FDG-Tz *In Vivo* PET/CT Imaging

**Figure 5:**
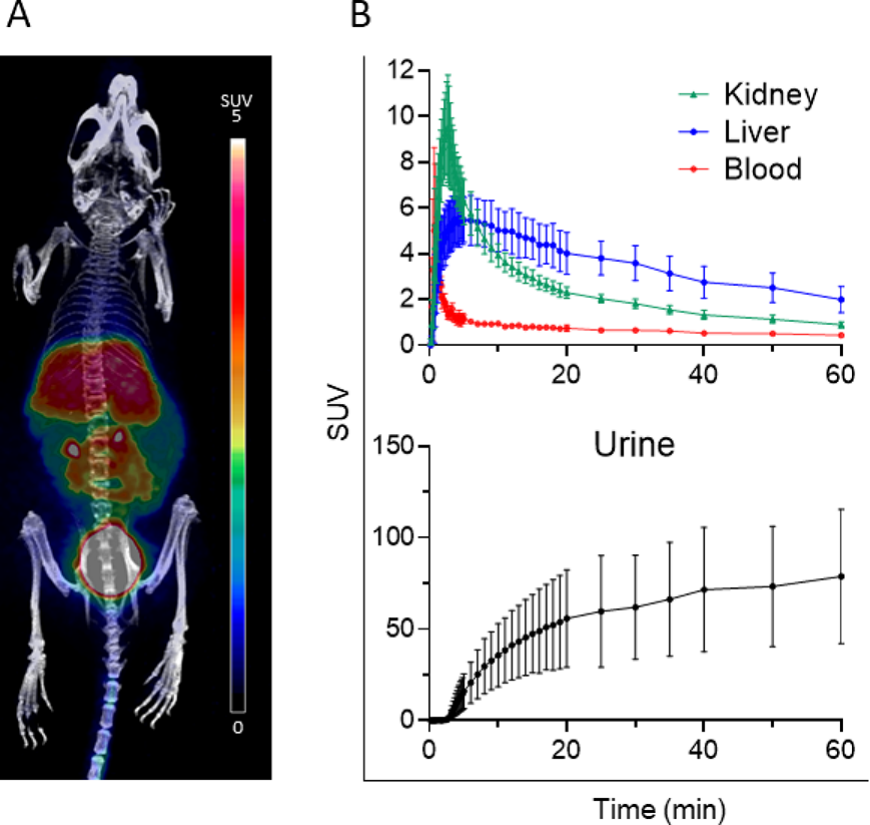
(A) Representative maximum intensity projection PET/CT image of a healthy mouse i.v. administrated with [^18^F]FDG-Tz (3.9 MBq). The image is a summation from 15–60 min postinjection. (B) Time–activity curves of selected tissues show rapid blood clearance, urinary excretion, and residual radioactivity in the liver and kidney.

To investigate [^18^F]FDG-Tz biodistribution, four healthy female Balb/cAnNrj mice were injected i.v. via the tail vein with 3.6–4.2 MBq of the tracer. Dynamic PET imaging showed moderate residual radioactivity in the liver at 60 min (SUV 2.00 ± 0.57 compared with 5.63 ± 1.11 at 4.5 min). Radioactivity was quickly eliminated from the blood (SUV 5.26 ± 2.69 at 0.8 min and decreased to 0.43 ± 0.06 at 60 min) and heart (SUV was at 5.06 ± 4.25 at 0.6 min and decreased to 0.41 ± 0.05 at 60 min.). Brain SUV_peak_ was only 0.33 ± 0.20 at 0.8 min and decreased to 0.05 ± 0.01 at 60 min (Figure 5). Thus, the *in vivo* metabolic profile of [^18^F]FDG-Tz demonstrated urinary excretion with signs of minor hepatobiliary elimination, quick clearance from blood, and only residual signal in the liver at 60 min postinjection, compared to previous studies with [^18^F]FDR-Tz.^25^

The obtained results support that [^18^F]FDG as a prosthetic group for tetrazine conjugation is useful compared to [^18^F]FDR. [^18^F]FDG introduction lowered the uptake in the abdominal region around 10 times at 60 min postinjection, making the tissue quantification in this region more reliable. It also decreases the residual liver uptake at 60 min postinjection by over 15%, reinforcing the urinary excretion as a primary elimination route and improving PET image contrast. As discovered GLUT1 affinity for [^18^F]FDG-Tz is lower compared to [^18^F]FDR-Tz, resulting in lower accumulation in GLUT1-rich organs,^58^ faster clearing from the blood, and an improved target-to-background ratio.

### Synthesis and Characterization of TCO-Modified MSNA on the C_60_-Azide Core

NH_2_-MSNA used as precursor for [^18^F]TCO-MSNA and pretargeted experiments was synthesized using a previously reported two-step protocol for controlled assembly of MSNAs.^59^ The degree of TCO-substitution was assessed using a fluorescent label prior to radiolabeling, and two different TCO conjugation degrees were investigated: 400 and 140% TCO-loaded MSNAs.

### Radiosynthesis of Preclicked [^18^F]TCO-MSNA

The click reaction between [^18^F]FDG-Tz and TCO-MSNA resulted in >97% yield, determined by radio-TLC of the reaction mixture, and the purity of the final product after ultrafiltration was >99% according to radio-size-exclusion chromatography (SEC) and radio-TLC.

### Pretargeted Experiment and Comparison with Preclicked [^18^F]TCO-MSNA Evaluation

**Figure 6:**
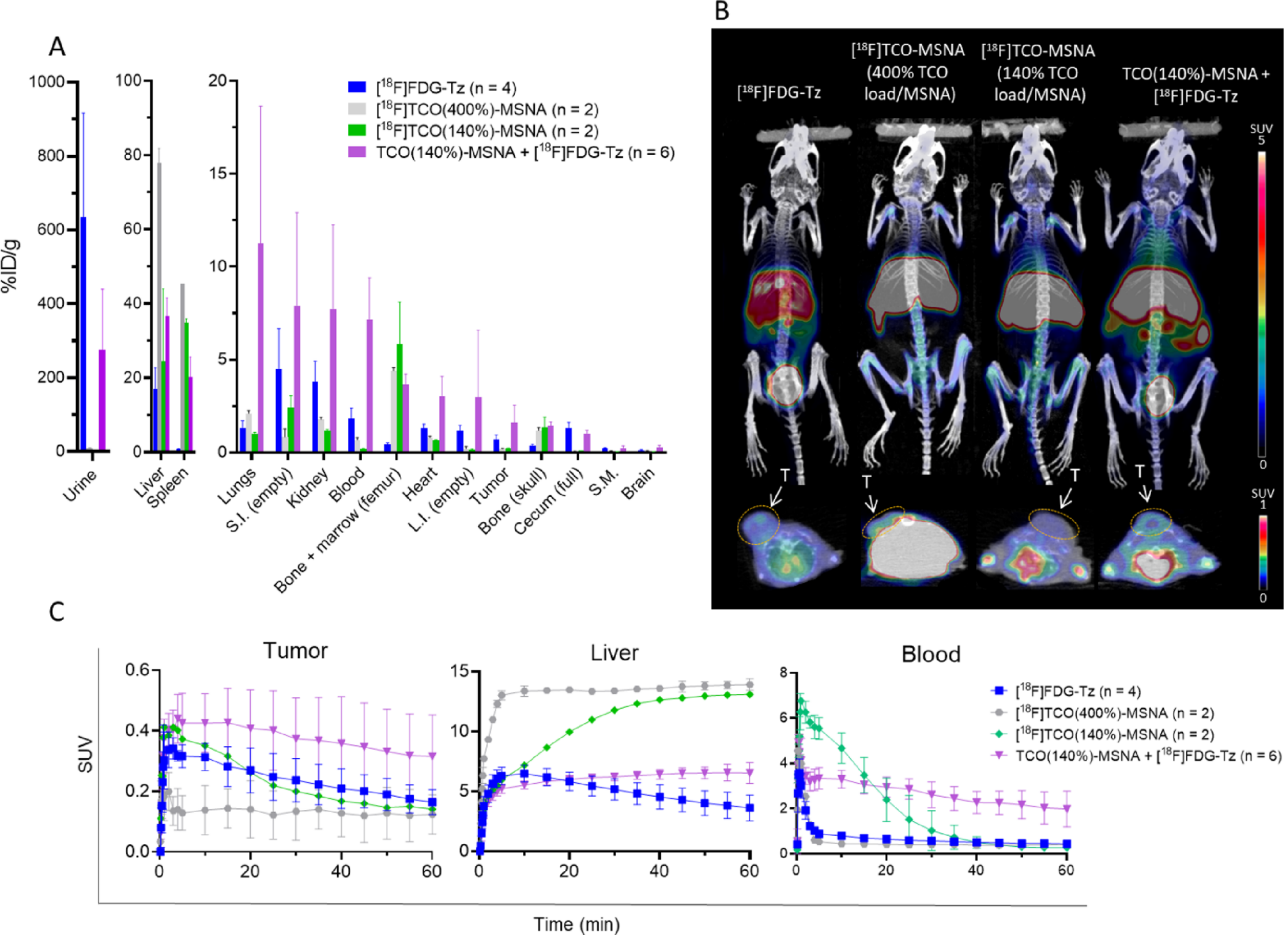
Biological evaluation of pretargeted TCO(140%)-MSNA injected 20 min prior injection of [^18^F]FDG-Tz and comparison to [^18^F]FDG-Tz and preclicked [^18^F]TCO(400%)-MSNA and [^18^F]TCO(140%)-MSNA in HCC1954 tumor-bearing female mice. (A) *Ex vivo* biodistribution at 60 min postinjection (S.M. – skeletal muscle, S.I. – small intestine, L.I. – large intestine). (B) Maximum intensity projection and axial plane PET/CT images at 15–60 min postinjection (*T* = tumor). (C) Time–activity curves of tumor, liver, and blood pool.

*In vivo* IEDDA reaction between [^18^F]FDG-Tz and TCO-MSNA was followed by PET imaging after i.v. administration of the radiotracer (5.4 ± 0.2 MBq) via the tail vein in HCC1954 tumor-bearing female mice 20 min after i.v. administration of TCO(140%)-MSNA. A 20 min lag time was selected for the pretargeted experiments to validate the occurrence of the bioorthogonal click reaction. The biological evaluation results were compared with [^18^F]FDG-Tz and the preclicked [^18^F]TCO-MSNA with different TCO load percentages (Figure 6, Figure S4). The pretargeted approach resulted in the highest uptake in the tumor among the studied tracers (SUV 0.32 ± 0.14 at 60 min postinjection), which was significantly higher than in animals treated only with [^18^F]FDG-Tz (SUV 0.16 ± 0.04, *p* = 0.04). In turn, preclicked [^18^F]TCO-MSNAs exhibited low tumor uptake, which could be caused by the differences in surface chemistry of the MSNAs, resulting in differences in their biodistribution, especially in blood. [^18^F]TCO-MSNA, with its completely [^18^F]fluoroglycosylated surface, was quickly harvested from the circulation, resulting in low radioactivity levels in blood immediately after 30 min postinjection (Figure 6C). While the pretargeted TCO-MSNAs, with partial *in vivo* [^18^F]fluoroglycosylation, exhibited high blood radioactivity levels even 60 min postinjection. As a result, the free TCO on the surface influenced the biodistribution in the pretargeted approach, leading to prolonged blood circulation and consequently higher tumor uptake compared to the preclicked approach.

Both *in vivo* and *ex vivo* experiments showed higher radioactivity levels in the blood for the pretargeted approach, and the TAC (time–activity curve) revealed slow elimination from the blood. In light of these results, a longer lag time between TCO-MSNA and [^18^F]FDG-Tz injections is needed for future studies. The blood SUV 1.97 ± 0.79 at 60 min postinjection was more than 7 times higher compared with other studied tracers. However, the pretargeted experiment stood out with favorably lower liver uptake compared to preclicked radiolabeled MSNA, e.g. SUV 6.6 ± 0.9 vs 13.91 ± 0.48 for [^18^F]TCO(400%)-MSNA at 60 min postinjection (*p* < 0.001).

As expected, the degree of TCO conjugation has an impact on the MSNA biodistribution. Two preclicked MSNA with 400 and 140% TCO loading/MSNA were investigated. According to *ex vivo* studies (Table S1) higher TCO percentage resulted in increased MSNA uptake in the blood, spleen, liver, lungs, and kidney 60 min postinjection.

## Conclusions

[^18^F]FDG-Tz was synthesized, and its biodistribution was evaluated in healthy mice to investigate the potential of this tracer as a pretargeted PET agent. The biological evaluation showed promising results, with quick elimination of [^18^F]FDG-Tz from blood and urinary excretion as the main elimination route. FDG as a carbohydrate moiety showed beneficial properties over the preciously investigated fluorodeoxyribose, FDR, due to its lower affinity to GLUT1. Despite the higher observed GLUT1 transportation of [^18^F]FDG-Tz inside cells *in vitro*, no significant transportation of [^18^F]FDG-Tz was observed in biodistribution experiments *in vivo*. The potential of [^18^F]FDG-Tz as a pretargeted PET agent was further confirmed in a pretargeted experiment with TCO-functionalized MSNA against HER2 in HCC1954 tumor-bearing mice. Pretargeted conditions revealed higher tumor uptake compared to the preclicked MSNA, but a longer lag time between TCO-MSNA and [^18^F]FDG-Tz is needed to avoid radiolabeling of the circulating TCO-MSNAs in blood.

## Experimental Section

### Reagents and Equipment

All chemicals were purchased from Sigma-Aldrich, Thermo Fisher, or Conju-Probe and used without further purification. Water was obtained from the Milli-Q purification system (Elga Purelab flex, UK). [^18^F]F^–^ for labeling was produced with the CC 18/9 cyclotron in a ^18^O(p,n)^18^F nuclear reaction. Radiosyntheses were conducted in the semiautomated device (DM Automation, Nykvarn, Sweden) in a shielded hot cell.

Chemical and radiochemical purity and stability of precursors and radiotracers were measured with analytical HPLC (Hitachi LaChrom Elite, Schaumburg, USA). Jupiter Proteo reversed-phase C18 column (4 μm, 250 mm × 4.6 mm) was used at a flow rate of 1 mL/min. HPLC eluents were A, 0.1% trifluoroacetic acid (TFA, v/v) in water; B, 0.1% TFA in acetonitrile. The gradient changed from 15% to 35% in 0–15 min. For radiolabeled MSNA quality control, SEC at a flow rate of 1 mL/min was used (Waters Protein-Pak 33SW (7.5 × 300 mm), eluent 0.1 M KH_2_PO_4_, pH 7.0).

TLC was performed with 95% acetonitrile in 5% water as eluent on TLC Merck Silica gel 60 F_254_ plates, which were exposed for 10 s against a phosphor imaging plate (BAS-TR2025, Fuji Photo Film Co. Ltd., Tokyo, Japan). Scanning was done with a FujiFilm FLA-5100 scanner (Fuji Photo Film Co. Ltd., Tokyo, Japan) and analyzed with AIDA Image Analyzer v.4.19 (Raytest Isotopenmessgeräte, Straubenhardt, Germany) at a resolution of 50 μm.

The purity of reference compounds was characterized by ^1^H and ^13^C nuclear magnetic resonance (NMR) with a Bruker 500 MHz spectrometer. Chemical shifts are presented in parts per million (ppm). The data is reported in the following order: chemical shift, multiplicity, coupling constant, and integration.

Syntheses of (*N*-(4-(1,2,4,5-tetrazin-3-yl)benzyl)-2-((((2*R*,3*R*,4*R*)-5-fluoro-2,3,4-trihydroxypentylidene)amino)oxy)acetamide) (FDR-Tz) and (*N*-(4-(1,2,4,5-tetrazin-3-yl)benzyl)-2-((((2*S*,3*S*,4*R*)-2-fluoro-3,4,5,6-tetrahydroxyhexylidene)amino)oxy)acetamide (FDG-Tz). Syntheses of reference compounds FDR-Tz and FDG-Tz were accomplished according to the previously reported methods.^25,26^ As previously reported, compound FDG-Tz comes on HPLC as three peaks for the acyclic *E*– and *Z*–, and the cyclic pyranose isomers (Figure S1A).

FDR-Tz: ^1^H NNMR (500 MHz, acetone) δ = 10.44 (s, 1H), 8.54 (d, *J* = 8.2 Hz, 2H), 8.12 (br, 0.19H), 7.83 (br, 0.88H), 7.65 (d, *J* = 7.0 Hz, 0.85H), 7.62 (d, *J* = 8.6 Hz, 2H), 6.96 (d, *J* = 6.5 Hz, 2H), 5.19 (q, *J* = 3.6 Hz, 0.21H), 4.78 (d, *J* = 5.9 Hz, 0.10H), 4.67–4.55 (m, 5H), 4.55 (s, 1H), 4.50–4.47 (m, 2H), 4.38 (d, *J* = 5.1 Hz, 0.37H), 3.92–3.85 (m, 1H), 3.78–3.76 (m, 1H), (Figure S5).

^13^C NMR (125.75 MHz, acetone) δ: 169.39, 166.21, 158.15, 153.47, 152.87, 144.92, 130.87, 128.26, 128.20, 127.99, 85.67, 84.34, 72.95, 72.90, 72.64, 71.12, 70.98, 70.32, 41.87, (Figure S6).

FDG-Tz: ^1^H NMR (500.08 MHz, DMSO) δ 10.57 (s, 1H), 8.53 (dt, *J* = 7.6 Hz, 1H), 8.46 (d, *J* = 8.4 Hz, 2H), 7.76 (t, *J* = 7.2 Hz, 1H), 7.55 (m, 3H), 7.17 (q, *J* = 5.3 Hz, 0.20H), 5.76 (dt, *J* = 12.4 Hz, 0.18H), 5.52 (d, *J* = 5.4 Hz, 0.23H), 5.24 (d, *J* = 6.7 Hz, 1H), 5.20 (d, *J* = 5.6 Hz, 1H), 5.14 (d, *J* = 6.7 Hz, 0.75H), 5.04 (dt, *J* = 12.4 Hz, 0.18H), 4.86 (d, *J* = 8.3 Hz, 0.17H), 4.61–4.57 (m, 4H), 4.49–4.45 (m, 2H), 4.43–4.39 (m, 1H), 4.17 (d, *J* = 9.1 Hz, 0.36H), 4.03–3.96 (m, 1H), 3.71–3.68 (m, 0.48H), 3.61–3.57 (m, 1H), 3.52–3.48 (m, 2H), (Figure S7).

^13^C NMR (125.75 MHz, DMSO-d6) δ: 170.49, 169.21, 165.90, 158.56, 149.38, 149.20, 145.10, 130.80, 128.45, 128.23, 91.87, 90.51, 78.67, 73.11, 71.44, 70.38, 70.21, 70.09, 70.06, 63.68, 61.32, 42.02, (Figure S8).

### Synthesis and Analysis of MSNA

TCO-functionalized MSNAs were prepared following a previously published protocol with minor modifications.^59^ Bicyclononyne (BCN)-modified HER2 mRNA targeting antisense phosphorothioate oligonucleotide (ON) sequence ON1 (Figure S9) was synthesized by an automated synthesizer using commercially available phosphoramidite building blocks and 3-phenyl 1,2,4-dithiazoline-5-one (POS) as a sulfurization reagent. The biological activity of the sequence in SNA formulation has previously been demonstrated.^42^ Synthesized ON1 was conjugated via strain-promoted alkyne–azide cycloaddition with an azide-modified [60]fullerene core (4 equiv) in DMSO overnight in rt, which gave C_60_-ON-conjugate C1 in 66% isolated yield after RP-HPLC purification (Figure S10). The authenticity of C1 was verified by MS (ESI-TOF). Then, the C_60_-ON-conjugate was exposed to excess (1.2 equiv/arm) of BCN-ON ON1 in aqueous 1.5 M NaCl solution. Incubation for 3 days in r.t. resulted in amino-modified NH_2_-MSNA in 40% isolated yield. The homogeneity of NH_2_-MSNA was confirmed by polyacrylamide gel electrophoresis (PAGE). For TCO-functionalization, TCO-PEG_4_-NHS (100 equiv) ester was added to aqueous borate buffered (pH 8.5) mixture of NH_2_-MSNA. After gentle shaking for 4 h at r.t., the TCO-modified TCO-MSNA was isolated with centrifugal filtration in 91% yield. The homogeneity and authenticity of TCO-MSNA were verified by PAGE and size-exclusion chromatography-multiple angle light scattering (SEC-MALS, Figure S11). The TCO-loading of TCO-MSNA was determined via test reaction with 6-methyl-tetrazine-carboxyfluorescein, and ratio in absorbances at 260 and 492 nm was used to quantify the TCO-content.

### Radiosynthesis of [^18^F]FDG-Tz

The cyclotron produced [^18^F]fluoride, dissolved in ^18^O-enriched water (Rotem Industries Limited, Arava, Israel), was trapped in a Sep-Pak QMA Plus Light cartridge (Waters, preconditioned with 10 mL of 0.5 M K_2_CO_3_, 15 mL of water). [^18^F]F^–^ was eluted from the cartridge with 2 mL of QMA-eluent solution (stock solution contains 237.5 mg of Kryptofix 2.2.2, 42.5 mg of potassium carbonate, 2 mL of Milli-Q water in 50 mL of acetonitrile). The reaction mixture was evaporated to dryness with Ar gas flow (70 mL/min for 10 min, and then 80 mL/min for 5–10 min more) under heating at 120 °C. The reaction vial was cooled to 50 °C with compressed air, and 1,3,4,6-tetra-*O*-acetyl-2-*O*-trifluoromethanesulfonyl-β-d-mannopyranose (25 mg, 52 μmol) dissolved in 0.5 mL of acetonitrile and 0.1 mL of thexyl alcohol (2,3-dimethylbutane-2-ol)) were added. The reaction mixture was heated at 85 °C for 5 min in the sealed vial. After cooling down to 25 °C, the reaction mixture was diluted with 0.8 mL of water and injected into semi-preparative HPLC (Jupiter Proteo column 4 μm, 250 mm × 10 mm, eluent A: water, eluent B: acetonitrile, flow rate 4 mL/min, 22–56% solvent B during 20 min; retention time was 14.5 min). The isolated protected intermediate was collected in a 50 mL bottle with 30 mL of water. After shaking, the mixture was passed through a tC18 Plus Light Cartridge (Waters, preconditioned with 10 mL of ethanol, 10 mL of water). The deprotection took place in the cartridge by passing through with 200 μL of 2 M NaOH. To neutralize the excess amount of sodium hydroxide, the receiving vial was preloaded with 25 μL of 6 M HCl. The volume of obtained [^18^F]FDG-Tz was approximately 250 μL. For oxime formation, tetrazine oxyamine HCl salt (0.57 mg, 1.9 μmol in 37.5 μL of water), 40 μL of methanol, and 80 μL of anilinium acetate buffer (1.2 M, pH 4.6) were added. The reaction was kept at 75 °C for 30 min, after which 0.9 mL of water was added, and the reaction mixture was injected to semi-preparative HPLC (Jupiter Proteo column 4 μm, 250 mm × 10 mm, eluent A: 0.1% TFA in water, eluent B: 0.1% TFA in acetonitrile, flow 4 mL/min, 12–22%, 15 min). The peaks with retention times of 19.2 and 20.1 min containing [^18^F]FDG-Tz were collected in a bottle with 25 mL of water, and after shaking, the mixture was passed through two connected C18 Plus Light Cartridges (Waters, preconditioned with 5 mL of ethanol, 10 mL of water). The product was eluted with 0.5 mL of absolute ethanol to a preheated conical reaction vial at 80 °C and kept for 5 min to reduce the ethanol amount by evaporation. The residue was cooled down to 40 °C, the final product [^18^F]FDG-Tz was formulated in phosphate-buffered saline (PBS), and the ethanol content was ≤10%. [^18^F]FDG-Tz was characterized by radio-HPLC with coinjection of the reference compound FDG-Tz (*R*_t_ = 10.8 min). The stability of [^18^F]FDG-Tz was analyzed by analytical radio-HPLC up to 6 h after EOS in the end product formulation at r.t.

### Preclicked Radiosynthesis of [^18^F]TCO-MSNA

To synthesize preclicked [^18^F]TCO-MSNA, 80 μL of PBS was added to [^18^F]FDG-Tz after the ethanol evaporation step. Part of this solution was taken and mixed with TCO-MSNA (1:2 to 1:4 ratio) and left at r.t. for 5 min. TCO-MSNA with 400 and 140% TCO load/MSNA were used for the reaction. The reaction mixture was ultrafiltrated (14100 × *g* for 3 × 5 min) and formulated into 0.01 M RNase-free PBS (pH 7.4). Quality control for the final product was done with radio-SEC and radio-TLC.

### Cell Cultures

CAL27 squamous cell carcinoma cells (ATCC CRL-2095) were purchased from the American Type Culture Collection (ATCC, Manassas, VA, USA). CAL27 cells were cultured in Dulbecco’s modified Eagle medium (DMEM; Gibco, ThermoFisher Scientific, Waltham, MA, USA) supplemented with l-glutamine (2.0 mM; ThermoFisher Scientific, Waltham, MA, USA), heat-inactivated fetal bovine serum (10%; Gibco, ThermoFisher Scientific, Waltham, MA, USA), and penicillin (50 U/mL)–streptomycin (50 μg/mL) solution (ThermoFisher Scientific, Waltham, MA, USA). CAL27 cells (passages 6–10) were seeded at the density of 5 × 10^5^ cells/wells onto 24-well plates. The cells were used in affinity and uptake studies 2 days after seeding. The culture medium was removed, and the cells were washed with prewarmed Hank’s balanced salt solution (HBSS) without glucose (HBSS; 125 mM NaCl, 4.8 mM KCl, 1.2 mM MgSO_4_, 1.3 mM KH_2_PO_4_, 1.3 mM CaCl_2_, and 25 mM HEPES adjusted to pH 7.4). The cells were then incubated with HBSS at 37 °C for 10 min before the experiments.

### Ability of Compounds to Compete for GLUT1-Binding with d-[^14^C]-glucose in CAL27 Cells

The GLUT1-binding ability of the studied compounds was performed by using a known radiolabeled GLUT1 substrate, d-[^14^C]-glucose (PerkinElmer, Waltham, MA, USA). Briefly, after preincubation with 500 μL of prewarmed glucose free HBSS, the cells were incubated at r.t. for 5 min with studied compounds (0.5–1400 μM) containing 1.8 μM (0.1 mCi/mL) of d-[^14^C]-glucose in glucose free HBSS (250 μL) and d-[^14^C]-glucose as blank. The reaction was stopped with ice-cold buffer, and the cells were washed two times with ice-cold buffer on the ice bath, lysed with 250 μL of 1% perchloric acid, and mixed with 1.0 mL of emulsifier safe cocktail (PerkinElmer, Waltham, MA, USA). The radioactivity was measured by a liquid scintillation counter (MicroBeta^2^ counter, PerkinElmer, Waltham, MA, USA), (Figure S12). Half-maximal inhibitory concentration (IC_50_) was calculated by nonlinear regression analysis.

### Concentration-Dependent Cell Uptake of Compounds in CAL27 Cells

The cells were first preincubated as described above. The cell uptake of these novel compounds was studied by adding 5–400 μM of studied compounds in 250 μL of prewarmed glucose free HBSS buffer on top of the cell layer and incubating the cells at r.t. for 5 min, based on the optimal performance of D-[^14^C]-glucose under these conditions. After incubation, the reaction was stopped with ice-cold buffer; the cells were then washed and lysed as described above.

For the LC-MS/MS analysis, an aliquot was taken from the lysates and diluted with acidified acetonitrile (0.1% formic acid) 1:1, including internal standard (IS). FDG-Tz was used as an internal standard for FDR-Tz, and vice versa. After protein precipitation, the samples were centrifuged for 10 min with 10,000 × *g* at 4 °C. The supernatant was then collected and transferred to vials for LC-MS/MS analysis.

The samples were analyzed with LC-MS/MS coupled with an Agilent 1200 series Rapid Resolution LC System (Agilent Technologies, Waldbronn, Germany) and Agilent 6410 Triple Quadrupole with an electrospray ionization (ESI) (Agilent Technologies, Palo Alto, CA, USA). The samples were injected (5 μL) into the reversed-phase HPLC column (Zorbax Eclipse XDB-C18 Rapid Resolution 4.6 × 50 mm, 1.8 μM, Agilent Technologies, Palo Alto, CA, USA). The aqueous mobile phase was 0.1% formic acid in water (A), while the organic mobile phase was 0.1% formic acid in acetonitrile (B).

The column temperature was 40 °C and the flow rate of 0.5 mL/min was used, with the following gradient 0–4 min: 5% → 95% B, 4–6 min: 95% B, 6–6.1 min: 95% → 5% B, 6.1–8 min: 5% B. The following instrument optimizations were used: 300 °C sheath gas heat, 6.5 L/min drying gas flow, 25 psi nebulizer pressure, and 4000 V capillary voltage, respectively. Detection was performed by using multiple reaction monitoring (MRM) with the following transitions: *m*/*z* 425 → 261 for FDG-Tz and *m*/*z* 395 → 261 for FDR-Tz.

The lower limit of quantification (LLOQ) for both studied compounds was 5 nM. These LC-MS/MS methods were also selective, accurate (RSD < 15%) and precise (RSD < 15%) over the range 5.0–2500 nM, with good linearity (*R*^2^ > 0.993).

Three replicates were analyzed from each concentration, and the statistical analyses were performed using GraphPad Prism v.5.03 software (GraphPad Software, San Diego, CA, USA).

### Animal Studies

Animal studies were approved by the national Project Authorization Board in Finland (licenses: ESAVI/12132/04.10.07/2017 and ESAVI/8648/2020) and were carried out in compliance with EU Directive 2010/EU/63 on the protection of animals used for scientific purposes. Healthy female Balb/cAnNrj mice (9–14 weeks old, 19 weeks old for fasting studies) were used for experiments with [^18^F]FDG-Tz. Female Rj:Athymic-*Foxn1*^*nu/nu*^ mice (8–10 weeks old) bearing subcutaneous HER2-expressing HCC1954 tumors were used for pretargeted and control preclicked experiments with [^18^F]TCO-MSNA, detailed information for the mice preparation was described earlier.^59^ Animals were housed in individually ventilated cages under a 12 h light/dark cycle with *ad libitum* access to water and food unless otherwise stated. Animals were anesthetized with isoflurane (induction 4–5%, maintenance 1.5–2.5%, 0.4 L/min O_2_) and sacrificed by cervical dislocation after applying cardiac puncture for blood collection.

### [^18^F]FDG-Tz *In Vivo* PET/CT Imaging and Analysis

For *in vivo* [^18^F]FDG-Tz studies, four mice were injected with 4.0–4.8 MBq in 50–80 μL volume via the tail vein. Animals were imaged with Molecubes β-cube (PET) and X-cube (CT) (MOLECUBES NV, Ghent, Belgium) for 60 min and reconstructed into time frames 30 × 10, 15 × 60, 4 × 300 s, and 2 × 600 s. For pretargeted experiments as well as control imaging with preclicked [^18^F]TCO-MSNA, Inveon Multimodality PET/CT (Siemens Medical Solutions, Knoxville, TN, USA) was used to perform dynamic PET with 6 × 10, 4 × 60, and 11 × 300 s time frames. For pretargeted imaging, TCO-MSNA (50 μL, 2.5 nmol of TCO) was injected 20 min prior to [^18^F]FDG-Tz (63 ± 17 μL) administration via the tail vein. For all experiments two animals were imaged at the same time under isoflurane anesthesia. Mice were sacrificed immediately after imaging for *ex vivo* evaluation.

Image analysis was performed with Carimas software (version 2.10, Turku PET Centre, Turku, Finland). CT scans were utilized as an anatomical reference combined with the radioactivity signal from PET images. Regions of interest (ROIs) within the selected organs were defined in three dimensions. Blood pool results were obtained from the heart left ventricle cavity; tumor ROIs were drawn excluding the fluid-filled tumor core (Figure S13). Standardized uptake values (SUV) were calculated, corrected with the remaining radioactivity in the cannula, tail, and syringe, and decay-corrected to the time of injection.

### *Ex Vivo* Evaluation

For [^18^F]FDG-Tz *ex vivo* evaluation, the mice were sacrificed at 15 (*n* = 6), 30 (*n* = 4), and 60 min (n = 2) postinjection. [^18^F]FDG-Tz distribution at 15 min postinjection was also studied in animals after fasting (*n* = 3, 4 h fasting prior to injection, water available *ad libitum*). Radiolabeled compound was administrated via the tail vein (1.8–11.4 MBq in 50–170 μL). For all experiments involving MSNA *ex vivo* measurements were performed after 60 min PET/CT imaging.

After sacrifice, tissue samples from organs of interest were dissected and weighed. Radioactivity was measured with a gamma counter (Triathler 3″; Hidex, Turku, Finland); the percentage injected radioactivity dose per gram of tissue (%ID/g) was calculated, corrected with the remaining radioactivity in the cannula, tail, and the syringe and decay-corrected to the time of injection.

### Radioactivity Distribution in Blood Components after i.v. Injection of [^18^F]FDG-Tz in Mice

To measure the radioactivity binding to blood cells, the blood obtained by cardiac puncture was placed into a lithium heparin tube, mixed according to the manufacturer’s instructions, and placed on ice. The blood sample was centrifuged at 4 °C for 5 min at 2118 × *g*. The plasma was separated by pipetting, and blood cells and plasma radioactivities were measured with a 1480 Wizard gamma counter (PerkinElmer/Wallac, Turku, Finland).

To determine the radioactivity portion bound to plasma proteins, plasma was mixed with an equal volume of acetonitrile and centrifuged at 14000 × *g* for 2 min at r.t. The radioactivity of both the supernatant and the protein pellet was measured with Wizard.

To determine the portion of intact tracer in plasma, a sample of deproteinized plasma supernatant was diluted with 0.1% TFA in water and analyzed with radio-HPLC.^60^

### Pretargeted experiment

For the pretargeted imaging, TCO-MSNA with 140% load/MSNA (2.5 nmol of TCO in 50 μL) was injected into the mice via the tail vein. Twenty minutes after that, [^18^F]FDG-Tz (5.4 ± 0.2 MBq in 40–80 μL, 52.7–118 pmol) was injected i.v. *In vivo* PET/CT imaging and *ex vivo* measurements were performed as described above.

### Statistical Analysis

Statistical analysis was done with GraphPad Prism 9 software. The SUV and %ID/g results are presented as mean ± standard deviation. Statistical differences between groups were determined by an unpaired multiple *t* test, and *p* < 0.05 was considered statistically significant.

## Supporting Information Available

Synthesis of [^18^F]FDG-Tz; NMR of FDG-Tz and FDR-Tz; radio-HPLC and radio-TLC of [^18^F]FDG-Tz; MSNA characterization; *ex vivo* biodistribution and statistical analysis; and time–activity curves (PDF)
