# Supplementary material for: Tetrazine Glycoconjugate for Pretargeted Positron Emission Tomography Imaging of trans-Cyclooctene-Functionalized Molecular Spherical Nucleic Acids
Source: ACS Omega. 2023 Nov 24;8(48):45326–36. doi: 10.1021/acsomega.3c04041 (PMC10702189; doi:10.1021/acsomega.3c04041)
Supplement: Supplementary file 1 — ao3c04041_si_001.pdf [file ao3c04041_si_001.pdf]

Supporting information

Tetrazine glycoconjugate for pretargeted PET imaging of trans-cyclooctene-functionalized molecular spherical nucleic acids

Tatsiana Auchynnikava[a,b], Antti Äärelä[b,c], Heidi Liljenbäck[a,d], Juulia Järvinen[e], Putri Andriana[a], Luciana Kovacs[a,b], Jarkko Rautio[e], Johan Rajander[f], Pasi Virta[b], Anne Roivainen[a,d,g], Xiang-Guo Li[a,b,g], Anu J. Airaksinen[a,b]*.

[a]Turku PET Centre, University of Turku, FI-20520 Turku, Finland.

[b]Department of Chemistry, University of Turku, FI-20500 Turku, Finland.

[c]Research and Development, Orion Pharma, FI-20380 Turku, Finland.

[d]Turku Center for Disease Modeling, University of Turku, FI-20520 Turku, Finland.

[e]School of Pharmacy, University of Eastern Finland, FI-70210 Kuopio, Finland.

[f]Accelerator Laboratory, Åbo Akademi University, FI-20520 Turku, Finland.

[g]InFLAMES Research Flagship Center, University of Turku, FI-20520 Turku, Finland.

* Corresponding author: Anu J. Airaksinen, email: anu.airaksinen@utu.fi.

A
B
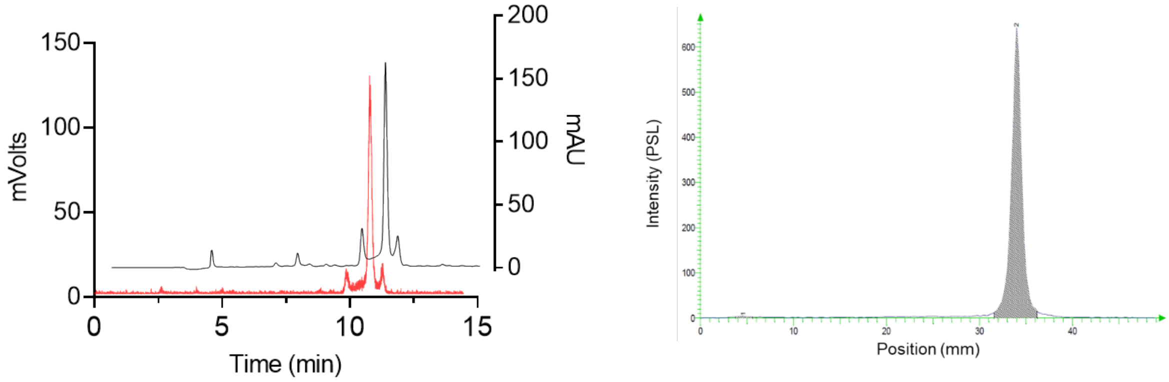

Figure S1. Quality control of final purified [^{18}F]FDG-Tz. (A) Radio-HPLC; (B) radio-TLC.

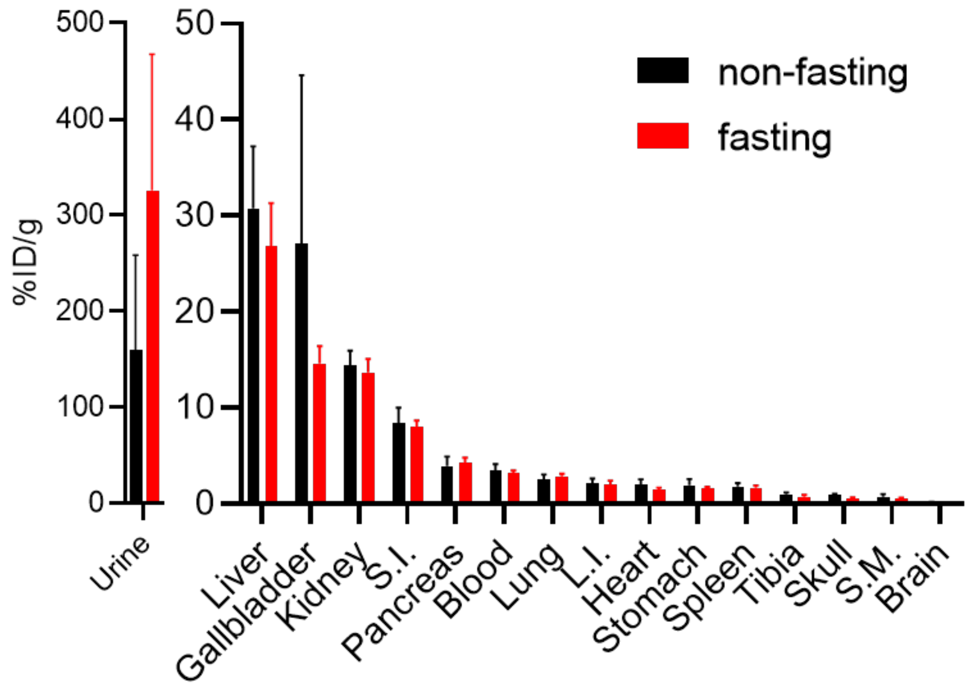

Figure S2. [^{18}F]FDG-Tz *ex vivo* biodistribution comparison between fasted (n = 3, 4 h prior to injection) and non-fasted (n = 6) mice 15 min postinjection, show no significant differences between the two conditions. (S.M. – skeletal muscle, S.I. – small intestine, L.I. – large intestine).

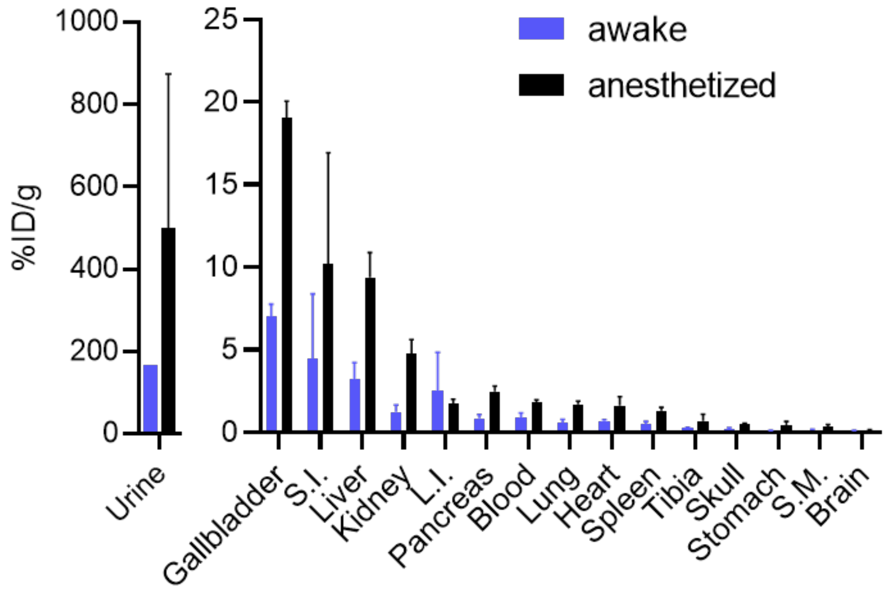

Figure S3. [^{18}F]FDG-Tz *ex vivo* biodistribution comparison between isoflurane anesthetized (n = 4) and awake mice (n = 2) 60 min after i.v. injection showed a trend of lower organ accumulation in awake animals. (S.M. – skeletal muscle, S.I. – small intestine, L.I. – large intestine).

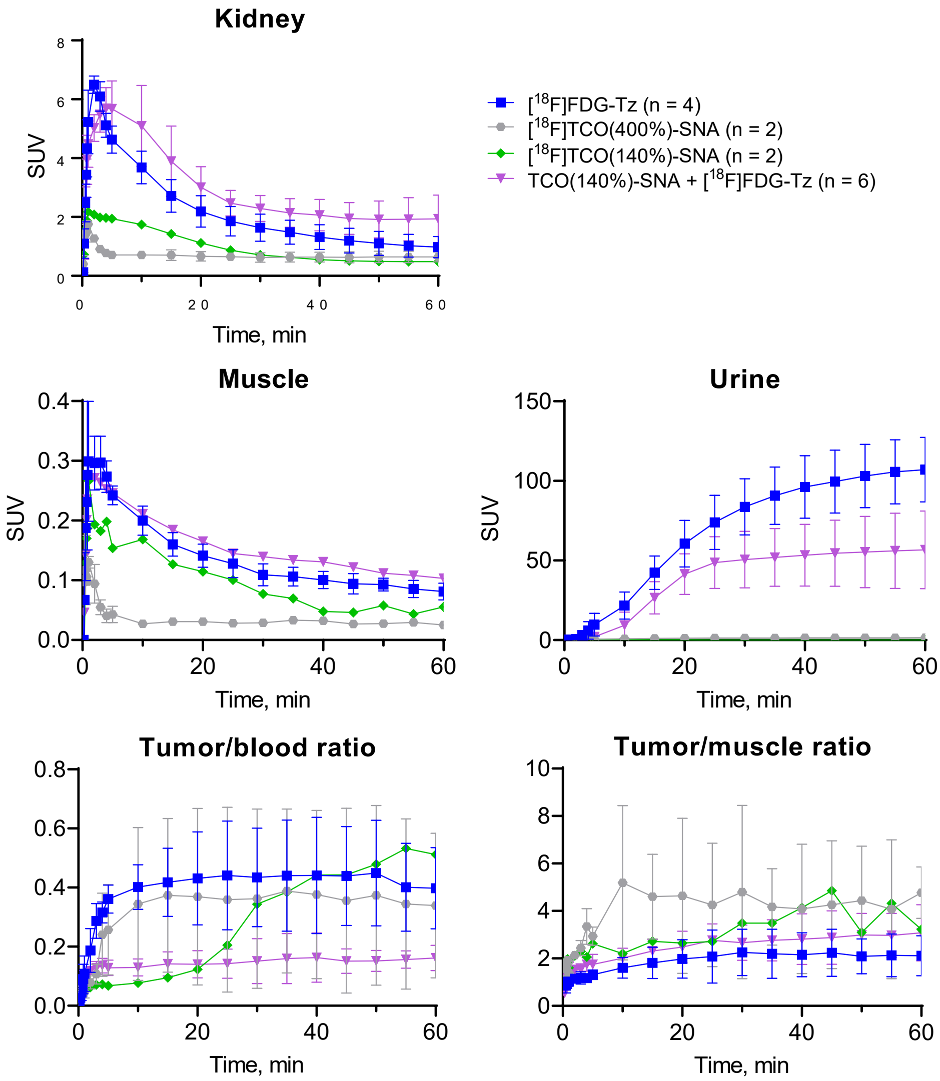

Figure S4. Time-activity curves of kidney, urine, muscle, tumor/blood ratio, and tumor/muscle ratio in HCC1954 tumor-bearing Rj:Athymic-*Foxn1*$^{nu/nu}$ mice with pretargeted TCO(140%)-SNA injected 20 min prior to injection of [^{18}F]FDG-Tz and compared to [^{18}F]FDG-Tz and pre-clicked [^{18}F]TCO(400%)-MSNA and [^{18}F]TCO(140%)-MSNA.

Table S1. *Ex vivo* biodistribution and statistical analysis in HCC1954 tumor-bearing female mice 60 min after administration of [^{18}F]FDG-Tz, [^{18}F]TCO(400%)-MSNA, [^{18}F]TCO(140%)-MSNA, or TCO(140%)-MSNA + [^{18}F]FDG-Tz.

**Tissue**	**%ID/g**				**Statistical significance (p-value)**
	**[^{18}F]FDG-Tz (n = 4)**	**[^{18}F]TCO(400%)-MSNA (n = 2)**	**[^{18}F]TCO(140%)-MSNA (n = 2)**	**TCO(140%)-MSNA + [^{18}F]FDG-Tz (n = 6)**	**[^{18}F]FDG-Tz vs. TCO(140%)-MSNA + [^{18}F]FDG-Tz**
Blood	1.83 ± 0.55	0.67 ± 0.13	0.20 ± 0.02	7.18 ± 2.22	** (0.0014)
Plasma	2.29 ± 0.83	0.09 ± 0.03	0.05 ± 0.00	17.90 ± 8.63	** (0.0066)
Urine	633.98 ± 283.06	7.93 ± 2.59	4.42 ± 0.30	276.60 ± 163.43	ns (0.0799)
Tumor	0.69 ± 0.26	0.18 ± 0.04	0.23 ± 0.00	1.62 ± 0.92	ns (0.0583)
Skin	0.70 ± 0.22	0.06 ± 0.00	0.18 ± 0.02	0.91 ± 0.21	ns (0.1738)
Lungs	1.32 ± 0.38	2.10 ± 0.16	0.99 ± 0.08	11.24 ± 7.42	* (0.0220)
Heart	1.30 ± 0.22	0.80 ± 0.06	0.68 ± 0.01	3.05 ± 1.03	** (0.0079)
Thyroid glands	1.68 ± 1.00	0.58 ± 0.14	0.50 ± 0.32	3.40 ± 1.37	ns (0.0664)
Pancreas	1.93 ± 0.45	0.14 ± 0.05	0.09 ± 0.04	1.54 ± 0.54	ns (0.2562)
Spleen	0.85 ± 0.16	45.44 (n = 1)	34.81 ± 1.10	20.32 ± 5.30	*** (0.0003)
Kidney	3.79 ± 1.12	1.78 ± 0.10	1.18 ± 0.05	7.73 ± 4.53	ns (0.0887)
Adrenal gland	0.73 ± 0.10	5.89 ± 0.18	6.92 ± 2.11	8.41 ± 2.06	** (0.0011)
Liver	17.11 ± 5.60	77.85 ± 3.92	24.47 ± 19.50	36.52 ± 5.04	** (0.0013)
Small intestine (empty)	4.51 ± 2.16	0.83 ± 0.43	2.41 ± 0.64	7.89 ± 5.03	ns (0.1879)
Large intestine (empty)	1.17 ± 0.28	0.19 ± 0.13	0.14 ± 0.04	2.99 ± 3.59	ns (0.2700)
Stomach (full)	0.63 ± 0.27	0.14 ± 0.08	0.19 ± 0.17	2.38 ± 1.65	* (0.0474)
Cecum (full)	1.30 ± 0.31	0.05 ± 0.00	0.08 ± 0.00	0.98 ± 0.21	ns (0.1319)
Feces	1.27 ± 0.89	0.02 ± 0.01	0.02 ± 0.00	0.52 ± 0.16	ns (0.1885)
White adipose tissue	0.32 ± 0.14	0.11 ± 0.07	0.12 ± 0.02	0.69 ± 0.38	ns (0.0700)
Ovaries	0.85 ± 0.20	2.50 ± 1.82	3.13 ± 0.17	3.55 ± 1.15	** (0.0019)
Uterus	0.95 ± 0.09	0.53 ± 0.05	0.58 ± 0.08	1.84 ± 1.03	ns (0.0886)
Muscle	0.24 ± 0.02	0.06 ± 0.02	0.03 ± 0.01	0.29 ± 0.11	ns (0.9937)
Salivary glands	0.48 ± 0.12	0.09 ± 0.00	0.09 ± 0.02	0.64 ± 0.24	ns (0.2277)
Lymph node	0.76 ± 0.19	0.41 ± 0.01	0.34 ± 0.09	1.57 ± 0.42	** (0.0039)
Bone (skull)	0.63 ± 0.14	1.18 ± 0.14	1.33 ± 0.58	1.44 ± 0.20	*** (0.0000)
Bone + marrow (femur)	1.19 ± 0.05	4.40 ± 0.16	5.83 ± 2.27	3.66 ± 0.57	*** (0.0000)
Brain	0.06 ± 0.01	0.09 ± 0.01	0.04 ± 0.00	0.26 ± 0.12	* (0.0261)
Urinary bladder	7.06 ± 7.23	0.32 ± 0.06	0.25 ± 0.06	5.96 ± 3.07	* (0.0218)

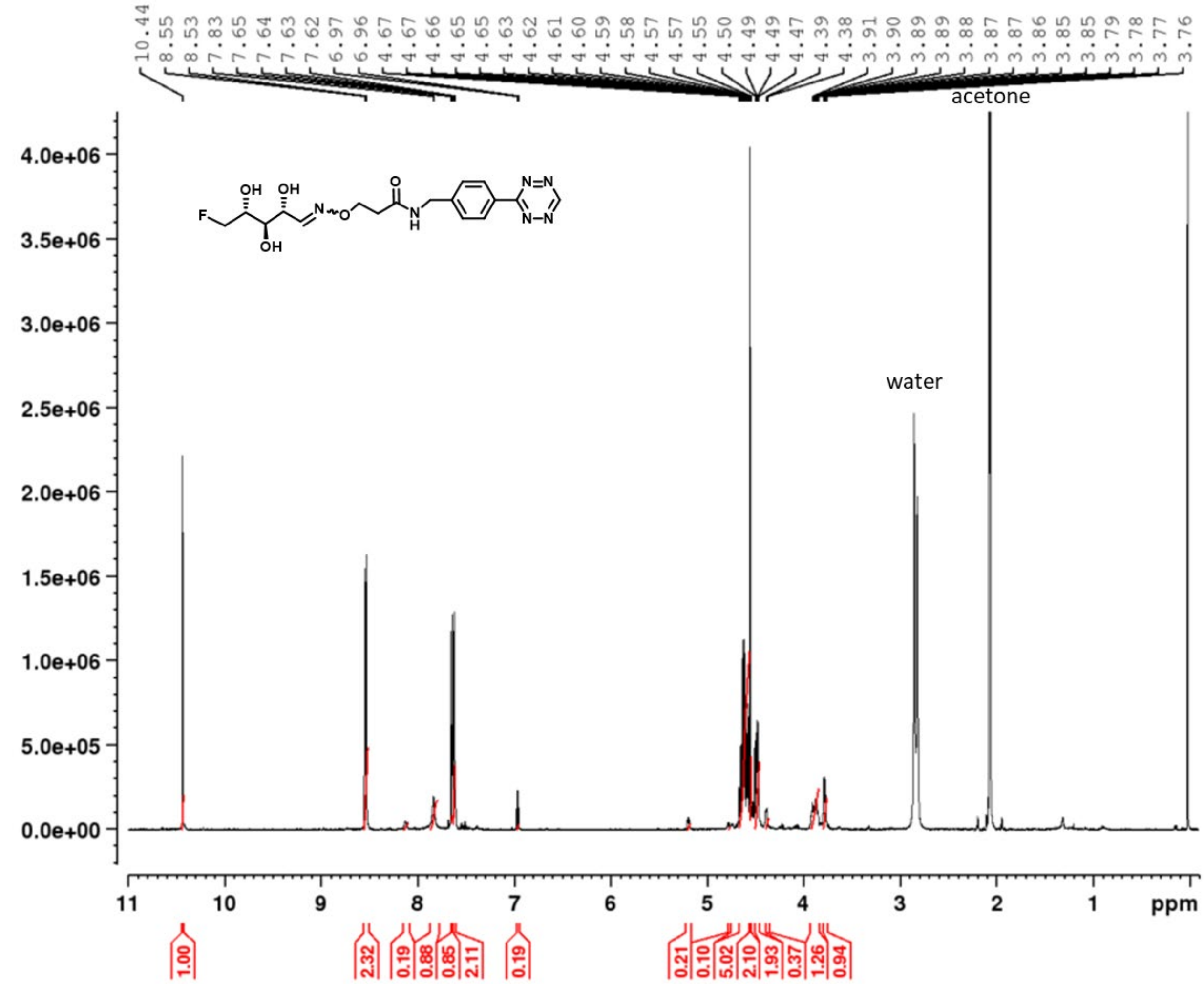

Figure S5. ^{1}H-NMR spectrum (500.08 MHz, acetone) of compound FDR-Tz.

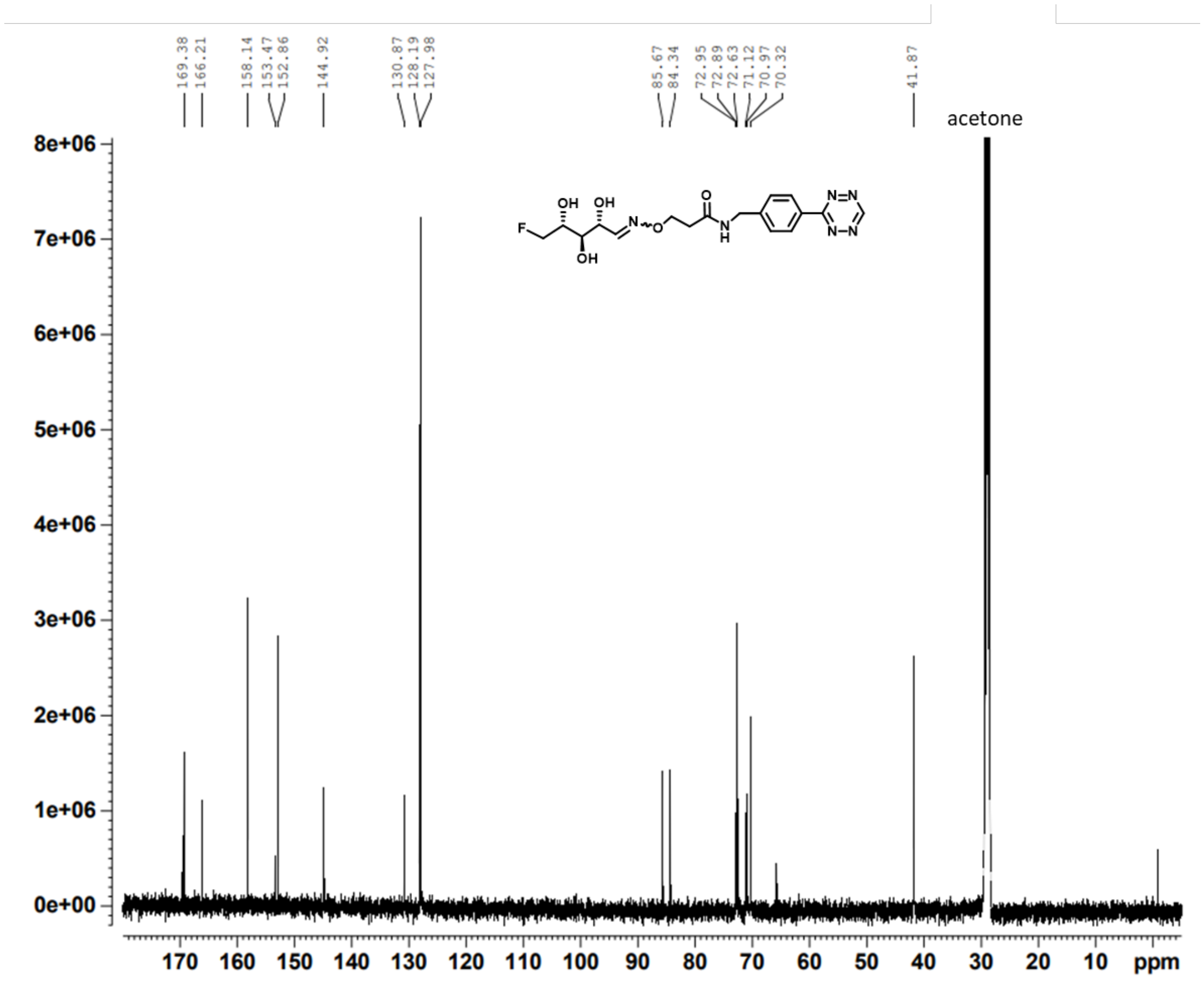

Figure S6. ^{13}C-NMR spectrum (125.75 MHz MHz, acetone) of compound FDR-Tz.

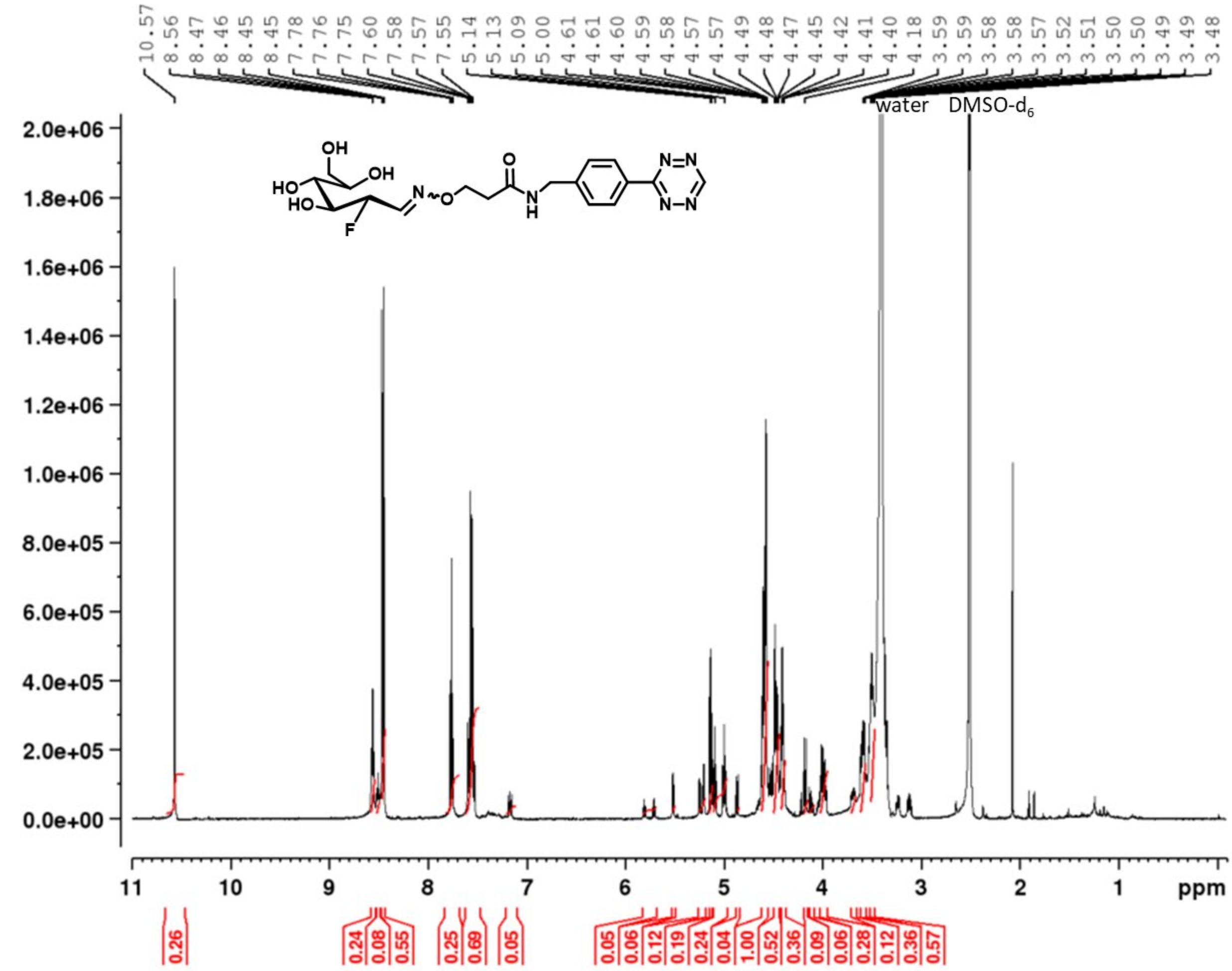

Figure S7. ^{1}H-NMR spectrum (500.08 MHz, DMSO-d$_6$) of compound FDG-Tz.

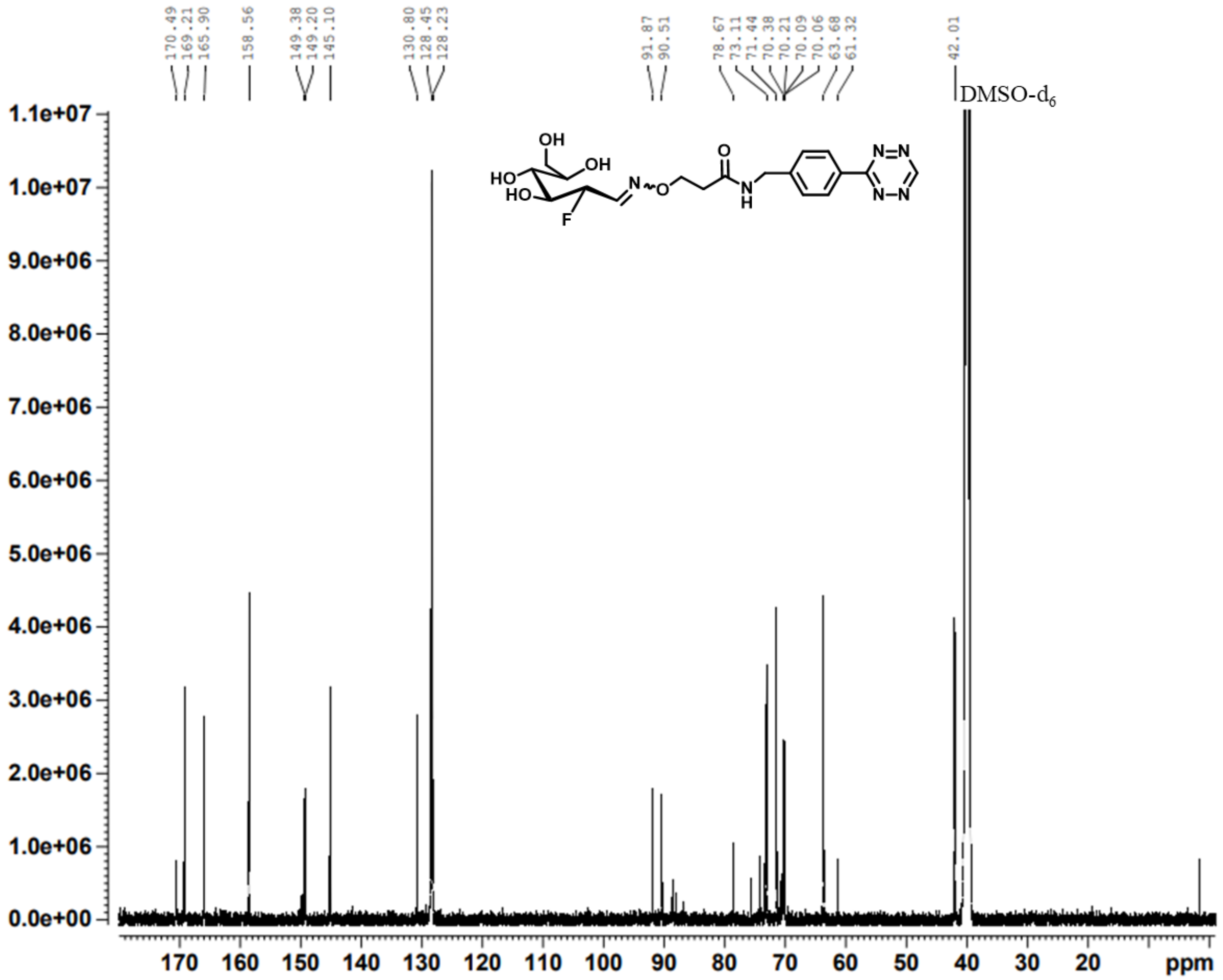

Figure S8. ^{13}C-NMR spectrum (125.75 MHz, DMSO-d_6) of compound FDG-Tz.

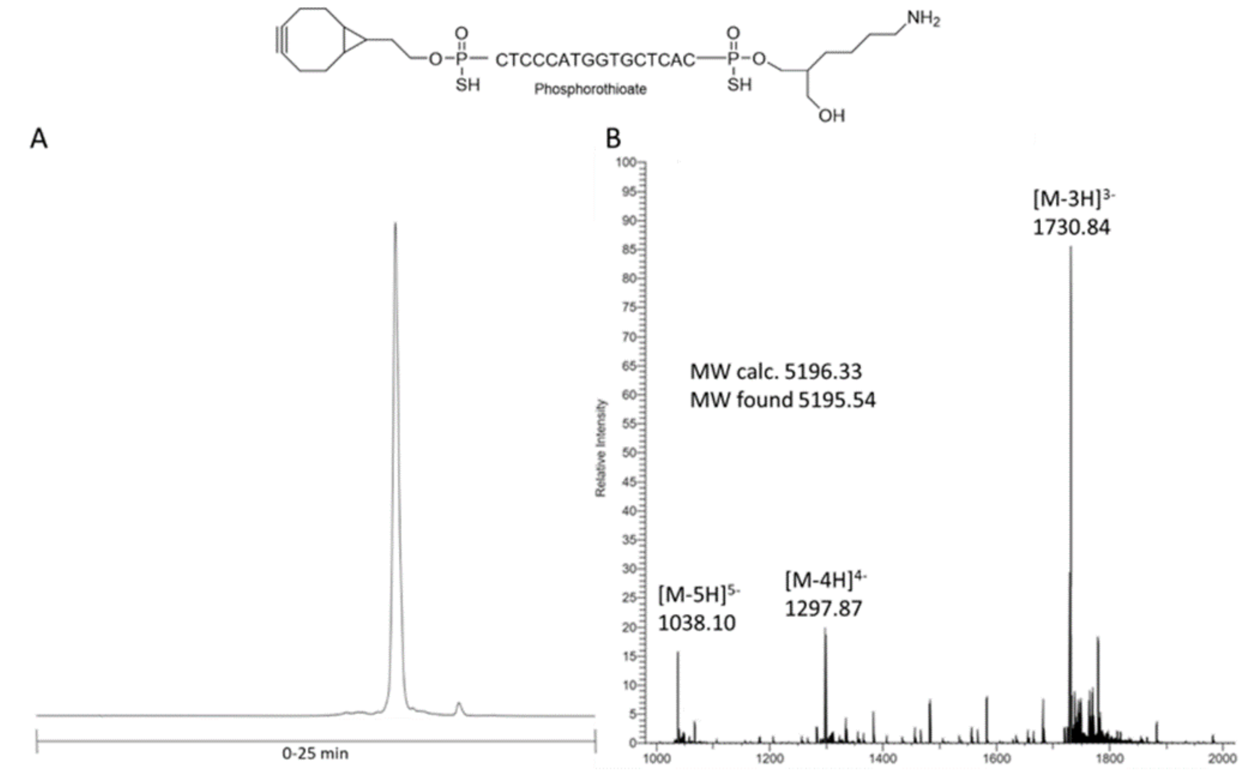

Figure S9. Structure and characterization of BCN-modified ON. (A) RP HPLC profile and (B) MS (ESI-TOF) spectrum. RP HPLC conditions: an analytical RP column (250 × 4.6 mm, 5 µm), detection at λ = 260 nm, gradient elution (0–25 min) from 5 to 45% MeCN in 50 mM triethylammonium acetate, flow rate 1.0 mL min^{-1}.

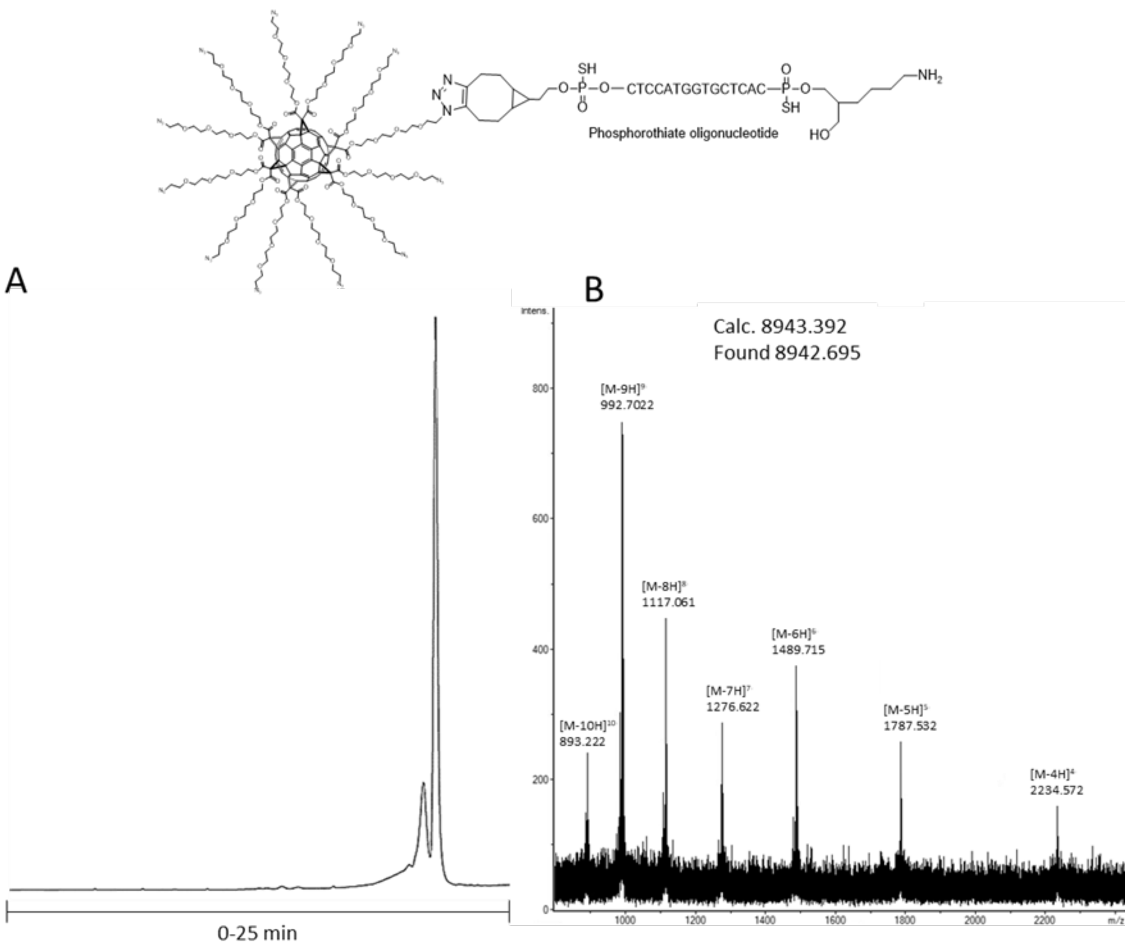

Figure S10. Structure and characterization of C60-ON conjugate **C1**. (A) RP HPLC profile and (B) MS (ESI-TOF) spectrum. RP HPLC conditions: an analytical RP column (250 × 4.6 mm, 5 µm), detection at λ = 260 nm, gradient elution (0–25 min) from 40% to 100% MeCN in 50 mM triethylammonium acetate, flow rate 1.0 mL min^{-1}.

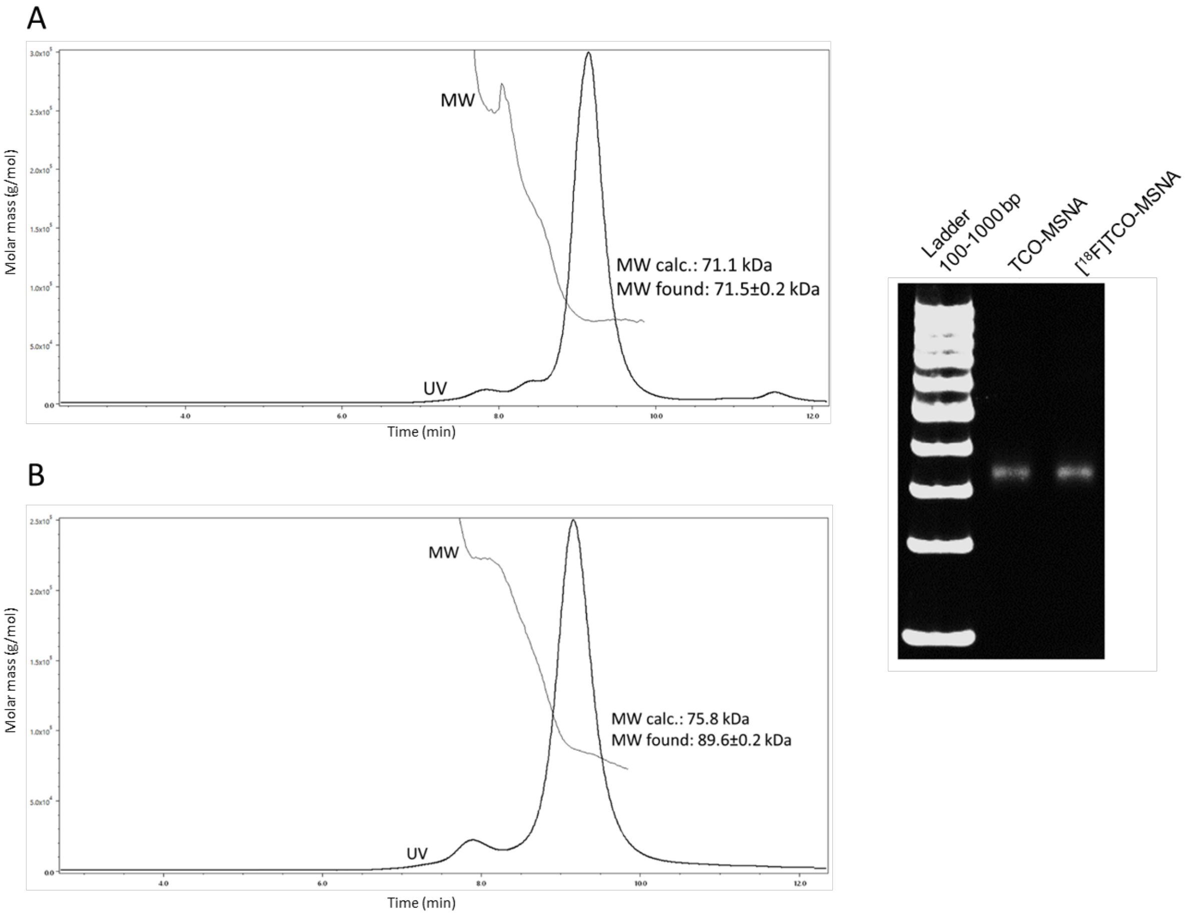

Figure S11. SEC-MALS profile of (A) TCO-MSNA, (B) [^{18}F]TCO-MSNA.

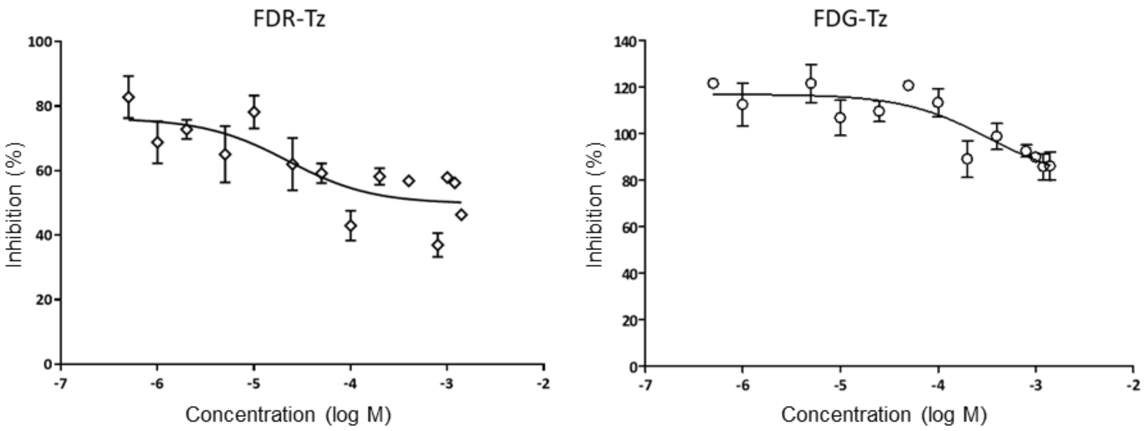

Figure S12. Inhibition of *D*-[^{14}C]glucose (1.8 µM) uptake in CAL27 cells in the presence of 0.5–1400 µM concentrations of studied compounds. IC_{50} for FDR-Tz is 22.4 µM, and IC_{50} for FDG-Tz is 324.4 µM.

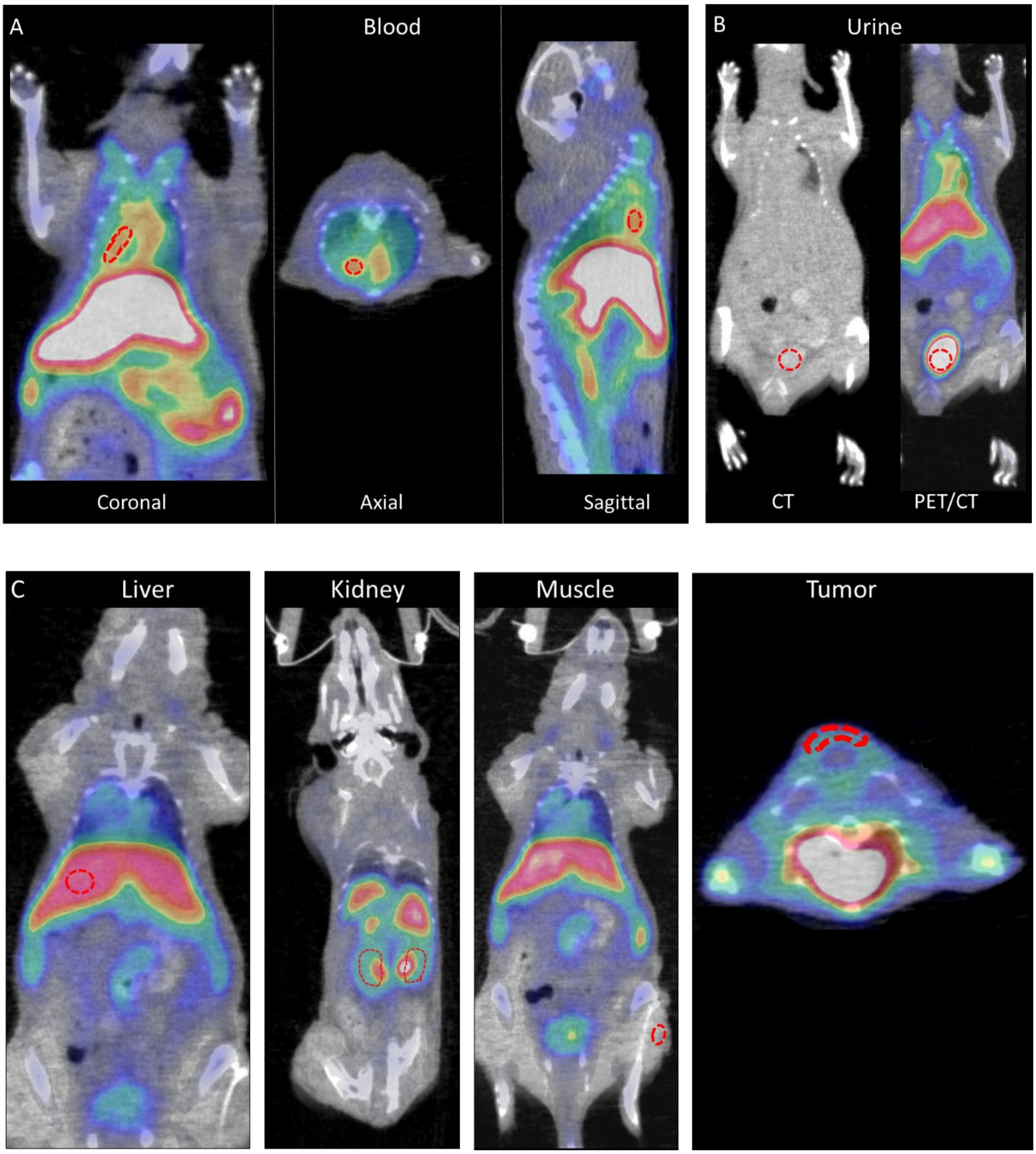

Figure S13. Representative PET/CT images of manually drawn regions of interest (ROIs) within selected organs. (A) All ROIs were defined in three dimensions as shown in this example for blood pool from the heart left ventricle cavity. (B) CT scans were utilized as anatomical reference combined with radioactivity signal from PET images as shown for urine ROI. (C) Liver ROIs were drawn as far away from heart and gallbladder signals as possible. ROIs were drawn for both kidneys. Muscle ROIs were drawn on the thigh region. Tumor ROIs were drawn excluding the fluid-filled tumor core.
